# LiNa_3_(SO_4_)_2_·6H_2_O: a lithium double salt causing trouble in the industrial conversion of Li_2_SO_4_ into LiOH

**DOI:** 10.1107/S2056989021008057

**Published:** 2021-08-10

**Authors:** Horst Schmidt, Iris Paschke, Wolfgang Voigt

**Affiliations:** a TU Bergakademie Freiberg, Institute of Inorganic Chemistry, Leipziger Str. 29, D-09596 Freiberg, Germany

**Keywords:** crystal structure, lithium sulfate, double salt hydrate, isomorphism

## Abstract

Lithium tris­odium bis­(sulfate) hexa­hydrate, LiNa_3_(SO_4_)_2_·6H_2_O was crystallized from aqueous solution at 298 K and the structure solved at different temperatures between 90 and 293 K. The structure is isomorphic with the corresponding molybdate and selenate double salt hydrate. It belongs to the non-centrosymmetric trigonal space group *R*3*c* (161). The temperature dependence of the lattice parameters has been determined. Further characterization by powder XRD and thermal analysis is reported.

## Abstract 2

The crystal structure of LiNa_3_(SO_4_)_2_·6H_2_O is discussed. In the context of the production of LiOH for batteries, the formation of the double salt should be avoided. Detection of its presence by means of XRD is important.

## Chemical context   

In the presently preferred process of LiOH production for batteries, an aqueous Li_2_SO_4_ solution is reacted with NaOH at temperatures well below 273 K (mostly at 268 K) for separating sodium sulfate in the form of its deca­hydrate (Glauber salt) according to the equation

Li_2_SO_4(aq)_ + 2 NaOH_(aq)_ → 2 LiOH_(aq)_ + Na_2_SO_4_·10H_2_O_(s)_

The sodium sulfate hydrate is removed and from the remaining solution, water is evaporated to crystallize LiOH·H_2_O. However, during cooling the solution from ambient temperature, the solution passes the stability field of LiNa_3_(SO_4_)_2_·6H_2_O, which extends from 271.3 to 321 K (Sohr *et al.*, 2017 ▸). Once formed, it will not disappear on further cooling. Rapid and reliable detection of its presence or absence by means of XRD is important. A powder diffraction pattern is available from the PDF database (Powder Diffraction File 33-1258, Inter­national Center for Diffraction Data), but no conclusive comment is attached regarding the conditions under which the material was obtained and prepared for powder XRD. It is known that the material loses its water of crystallization very easily. Therefore, in their careful thermodynamic study of the system Li_2_SO_4_–Na_2_SO_4_–H_2_O at 298 K, Filippov & Kalinkin (1989 ▸) did not make an attempt to isolate the double salt hydrate because of instability. Ji *et al.* (2015 ▸) include a figure of the PXRD pattern, but only in a mixture with anhydrous LiNaSO_4_. The growth of crystals under defined conditions and deriving the PXRD pattern from single-crystal structure analysis could resolve doubts about the PXRD pattern.

LiNa_3_(SO_4_)_2_·6H_2_O was first crystallized by Mitscherlich (1843 ▸) and later, preparative conditions were specified (Scacchi, 1867 ▸). Early crystallographic characterization is summarized by Groth (1908 ▸), where the cited paper of Traube (1894 ▸) is of particular inter­est, since he determined the correct polar point group 3*m* for this compound and the isomorphic compounds LiNa_3_(*M*O_4_)_2_·6H_2_O with *M* = S, Se, Mo, Cr. Even a mixed compound LiNa_3_{(SO_4_)_0.5_(CrO_4_)_0.5_} was described within this series. A first crystal structure of the molybdate was published by Klevtsova *et al.* (1988 ▸). Later, Kaminskii and co-workers grew large crystals of the molybdate (Kaminskii *et al.*, 2009 ▸) and selenate (Kaminskii *et al.*, 2007 ▸) for studies on the non-linear optical effects of the materials, where they also re-determined and refined the crystal structures at ambient temperature, but without discussion of structural details.

## Structural commentary   

**Figure 1:**
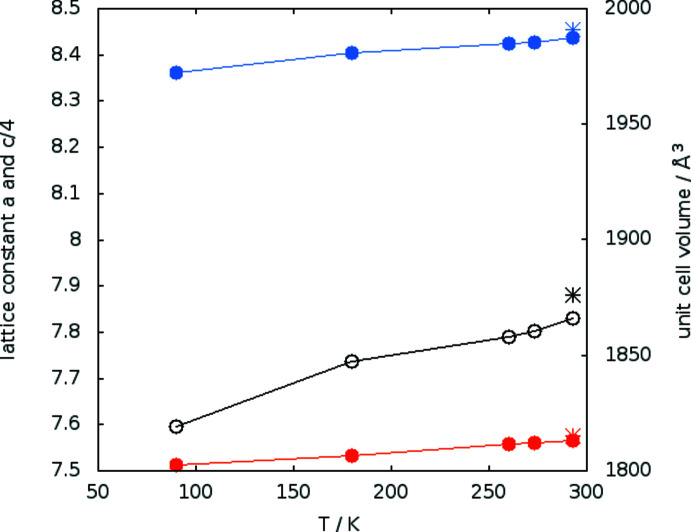
Variation of lattice parameters with temperature; *a* axis in red, *c* axis in blue, values divided by four, volume shown in black; circles: from single-crystal measurements, stars: data from powder X-ray measurement: *a* = 8.4552 (7) Å, *c* = 30.3032 (3) Å, *V* = 1876.18 Å.

**Figure 2:**
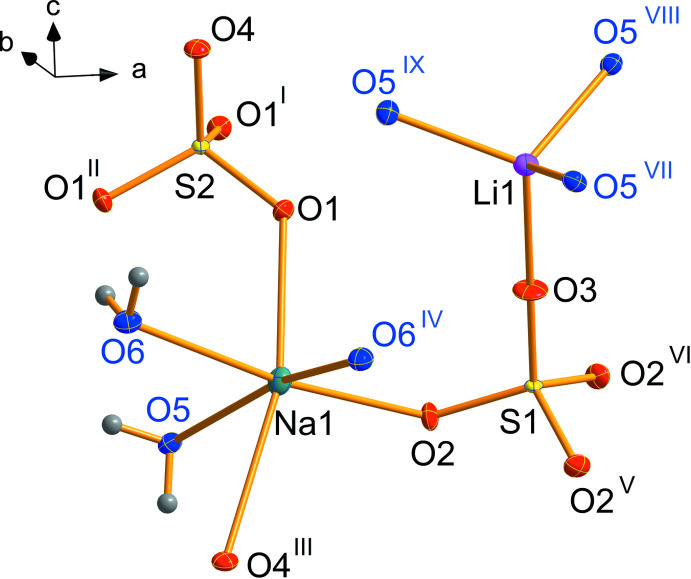
Asymmetric unit plus bonds. Ellipsoids are drawn at the 50% level. Symmetry codes: (I) 1 − *y*, 1 + *x* − *y*, *z*; (II) −*y* − *y*, 1 − *x*, *z*; (III) −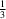 − *x* + *y*, −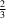 + *y*, −1/6 +) ; (IV) −*x* + *y*, −*x*, *z*; (V) 1 − *y*, *x* − *y*, *z*; (VI) 1 − *x* + *y*, 1 − *x*, *z*; (VII) 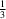 − *x* + *y*, −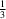 + *y*, 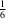 + *z*; (VIII) 4/3 − *y*, 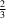 − *x*, 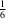 + *z*; (IX) 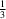 + *x*, 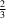 + *x* − *y*, 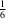 + *z*.

**Figure 3:**
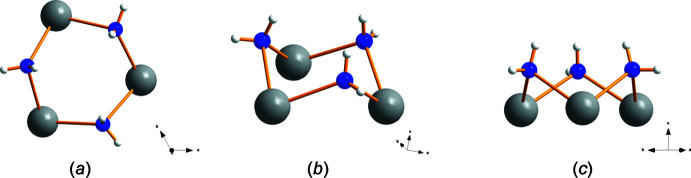
The trimeric unit Na_3_(H_2_O)_3_ viewing from different directions (*a*, *b* and *c*).

Single-crystal structure determination was performed at five temperatures between 90 and 293 K. At all temperatures, the structure could be solved in the polar space group *R*3*c* H (161). The cell parameters varied continuously with temperature (Table 1 ▸ and Fig. 1 ▸). Thus, the results confirm the isomorphism to the molybdate LiNa_3_(MoO_4_)_2_·6H_2_O (Kaminskii *et al.*, 2009 ▸) and no structural change within the investigated temperature range. Fig. 2 ▸ shows the asymmetric unit completed with atoms to visualize the coordination of sodium, lithium and sulfur. There is only one crystallographically distinguishable sodium and lithium position, but two for sulfur. Sodium is surrounded by six oxygen atoms, three belong to water mol­ecules (blue) and the remaining three to sulfate groups. The distance of 2.639 Å between Na1 and O4 is quite long. Also, the angle O1—Na1—O4 of 165° deviates considerably from 180°. However, in a first approximation the environment of sodium atoms can be described as a distorted octa­hedron. The water mol­ecules with O6 bridge three sodium ions to a trimeric unit as shown in Fig. 3 ▸. The trimers look like cyclo­hexane rings (Fig. 3 ▸ *b*) in a chair conformation with the water mol­ecules on the upper three points (Fig. 3 ▸ *c*).

**Figure 4:**
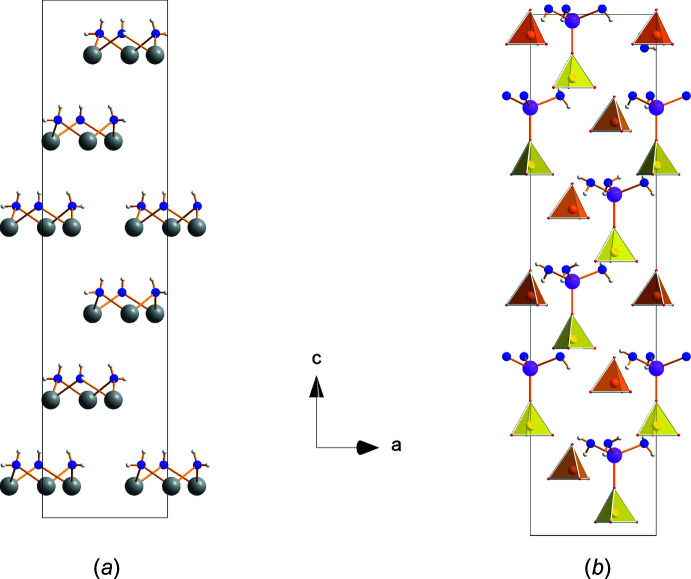
Stacking of (*a*) the trimeric Na_3_(H_2_O)_3_ and (*b*) the Li(H_2_O)_3_(SO_4_) units along the *c* axis within a unit cell. Additional SO_4_ groups with the S2 sulfur atom are shown (dark yellow).

The lithium cation is coordinated by three water mol­ecules (O5) and the apex (O3) of a sulfate anion containing S1 completes a tetra­hedron (Fig. 2 ▸). Thus, the trimeric Na_3_(H_2_O)_3_ and the double tetra­hedron Li(H_2_O)_3_(SO_4_) form the characteristic structural units of this compound. In Fig. 4 ▸, the arrangement of these units is shown within the unit cell separately for Na_3_(H_2_O)_3_ (Fig. 4 ▸ *a*) and Li(H_2_O)_3_(SO_4_) (Fig. 4 ▸ *b*). In Fig. 4 ▸ *b* the sulfate anions with S2 are added as darker colored tetra­hedra. The repeat unit requires stacking of six such units along the *c-*axis direction. The uniform orientation of the units underlines the polar character of the *c* axis.

## Supra­molecular features   

**Figure 5:**
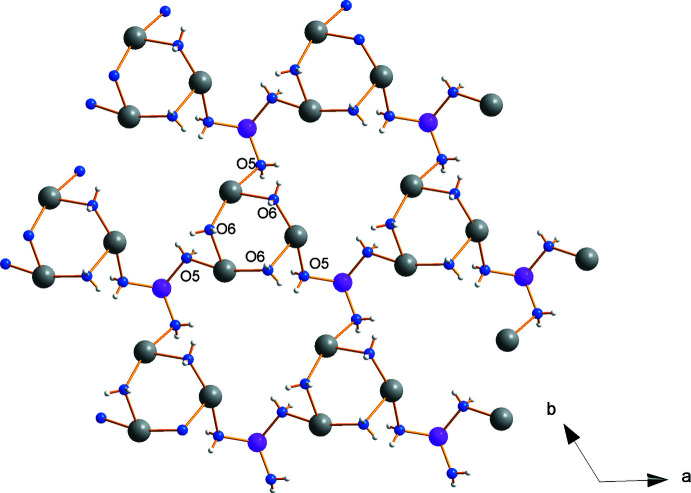
Cation–water coordination network within the *ab* plane. Sodium = gray, oxygen = blue, lithium = pink, hydrogen = white.

**Figure 6:**
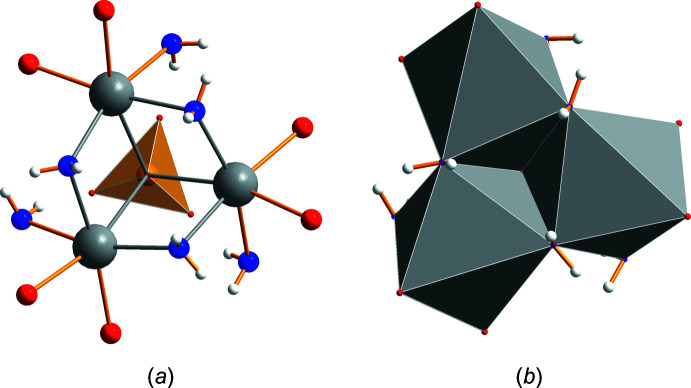
Representation of the sodium ion coordination within a trimeric unit: (a) stick and ball, (*b*) polyhedrons.

**Figure 7:**
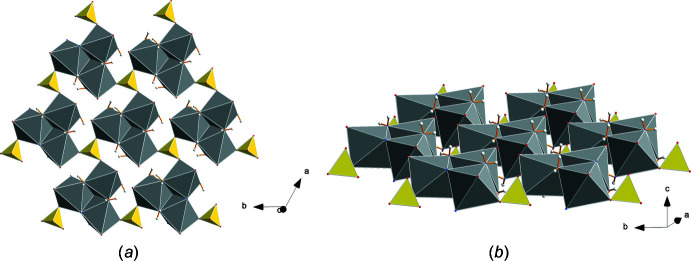
Inter­connection of trimeric units within one layer by sulfate tetra­hedra with the S1 sulfur atom viewed from two directions (*a* and *b*).

**Figure 8:**
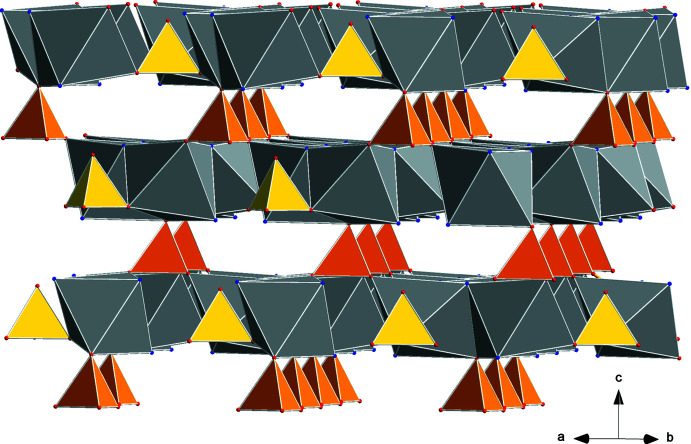
Inter­connections of sodium layers by sulfate tetra­hedra with the S2 sulfur atom (dark yellow).

**Figure 9:**
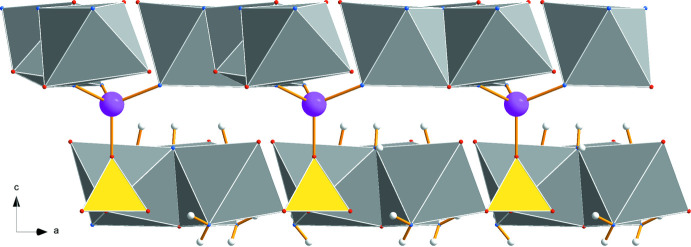
Inter­connections of sodium layers by sulfate tetra­hedra with the S2 sulfur atom (dark yellow).

The overall structure of the compound is polymeric with water and sulfate anions connecting the cations. The three water mol­ecules coordinated at the lithium cation are at the same time coordinated to three sodium cations, each sodium ion belonging to another trimeric sodium ring forming a water–cation coordination network, as shown in Fig. 5 ▸. When including the entire coordination spheres of sodium, one can describe the trimers as edge-bridged octa­hedra, as illustrated in Fig. 6 ▸ *a* and 6*b*. Thereby, the O4 oxygen from the sulfate anion of S2 represents a common coordination point from below (Fig. 6 ▸ *a*). The height of sulfur S1 along the *c* axis is near that of Na1. Thus, the three corners of this sulfate tetra­hedron connect three trimeric units within a sodium ion layer, as shown in Fig. 7 ▸ from two viewing angles. As shown in Fig. 6 ▸, the sulfate with S2 is positioned with its oxygen atom (O4) at the center below the trimeric units, and thus the other three O1 atoms of this sulfate anion connect three trimeric sodium units from the adjacent layer below (Fig. 8 ▸). In this way, the sulfate with S2 acts as a connector between sodium layers and the sulfate with S1 within one layer. Additional inter­connections between layers are realized by the sulfate of S1 as part of the double tetra­hedron Li(H_2_O)_3_(SO_4_), as illustrated in Fig. 9 ▸.

**Figure 10:**
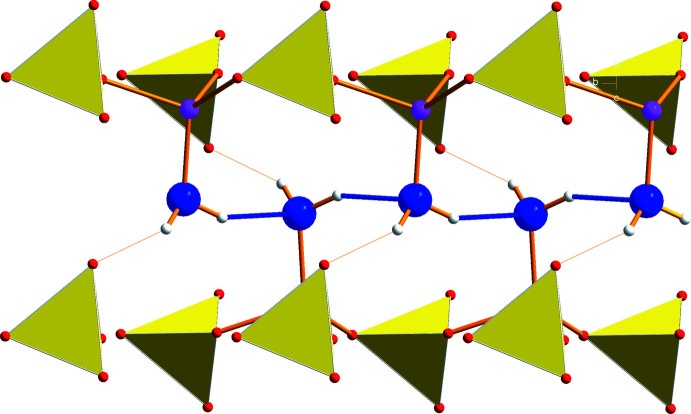
Hydrogen-bonded (blue bonds) chain of water mol­ecules along the *b-*axis direction in the structure of Li_2_SO_4_·H_2_O (Fugel *et al.*, 2019 ▸)

**Figure 11:**
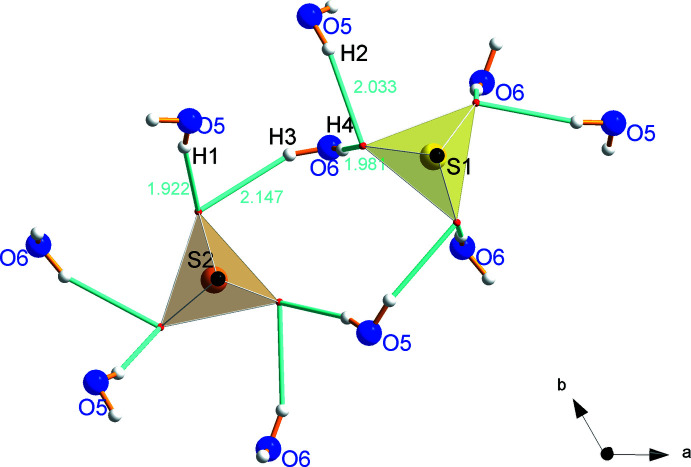
Hydrogen bonds from water mol­ecules to the S1 and S2 sulfate groups.

**Figure 12:**
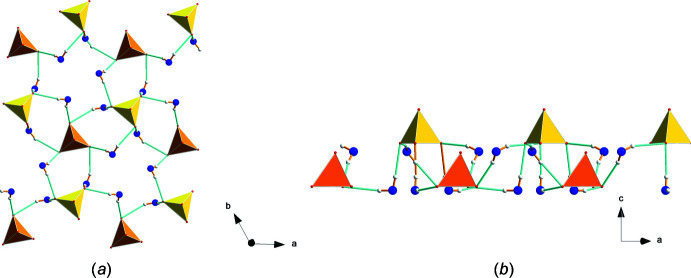
Larger part of the hydrogen-bond network projected on (*a*) the *ab* plane and (*b*) the *ac* plane.

Investigation of the hydrogen-bond network (Table 1 ▸) revealed that, inter­estingly, the water mol­ecules form hydrogen bonds only to the sulfate groups, but not between themselves as is observed in a channel-like arrangement in Li_2_SO_4_·H_2_O (Fig. 10 ▸). However, as can be seen from Fig. 11 ▸, the hydrogen atoms H1 and H3 share O1 as a common acceptor atom of the sulfate with S1, and H2 and H4 do the same with O2 at the sulfate anion of S2. The bond lengths vary between 1.92 and 2.15 Å. Fig. 12 ▸ shows a larger part of the hydrogen-bond network, projected both along the *c* axis (Fig. 12 ▸ *a*) and perpendicular to the *c* axis (Fig. 12 ▸ *b*). From the latter, it can be recognized that the hydrogen bonds contribute to the bonding strength within a layer, but not between the layers. Connections between the layers are established by cation–anion coordination as shown in Figs. 8 ▸ and 9 ▸.

## Database survey   

In the Inorganic Crystal Structure Database (ICSD), only 1164 records with space group *R*3*c* (No. 161) can be found. Most of them belong to the LiNbO_3_ or Whitlockite type [Whitlockite = MgCa_9_(PO_4_)_6_(HPO_4_)]. Compounds containing lithium in this space group numbered 179, of which 148 belong again to LiNbO_3_ type. The isomorphic molybdate (ICSD col 65006, col 420160) represents a structure type of its own. The isomorphic selenate LiNa_3_(SeO_4_)_2_·6H_2_O (Kaminskii *et al.* 2007 ▸) could not be found in the ICSD. Inter­estingly, the mineral chlorartinite, Mg_2_[Cl(OH)CO_3_]·2H_2_O, which forms easily in MgO-based building materials, also crystallizes in the space group *R*3*c* (Sugimoto *et al.*, 2006 ▸).

## Synthesis, crystallization and characterization   

**Figure 13:**
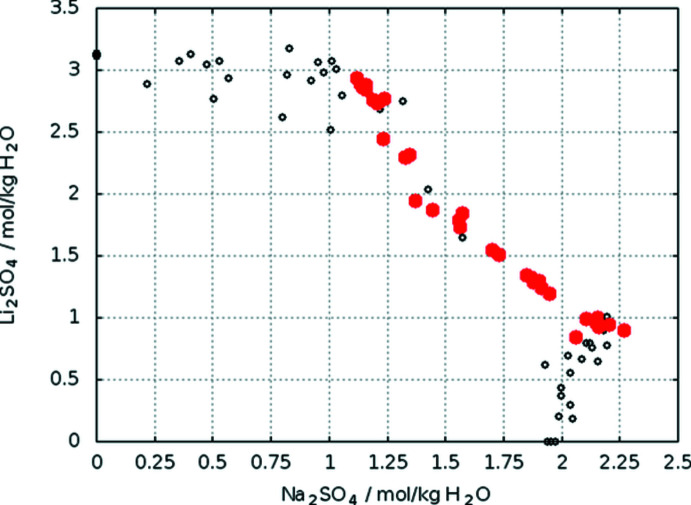
Solubility data in the system Li_2_SO_4_–Na_2_SO_4_–H_2_O at 298 K (Sohr *et al.*, 2017 ▸). Crystallization points of LiNa_3_(SO_4_)_2_·6H_2_O are shown in red.

Single crystals were grown from about 120 mL of an aqueous solution containing Li_2_SO_4_ and Na_2_SO_4_ in a molar ratio of approx. 1:1 and an absolute concentration well below the solubility line (Fig. 13 ▸). The solution was kept in an desiccator with 50% H_2_SO_4_ solution as drying agent. Over two weeks, a number of crystals with sizes of 1–7 mm were formed that showed the typical trigonal–pyramidal form. Small pieces were cut for XRD measurements. The density of 1.995 g cm^−3^ calculated from the parameters at 293 K (Table 1 ▸) is in excellent agreement with the experimental value of 2.009 g cm^−3^ as cited in Groth (1908 ▸).

**Figure 14:**
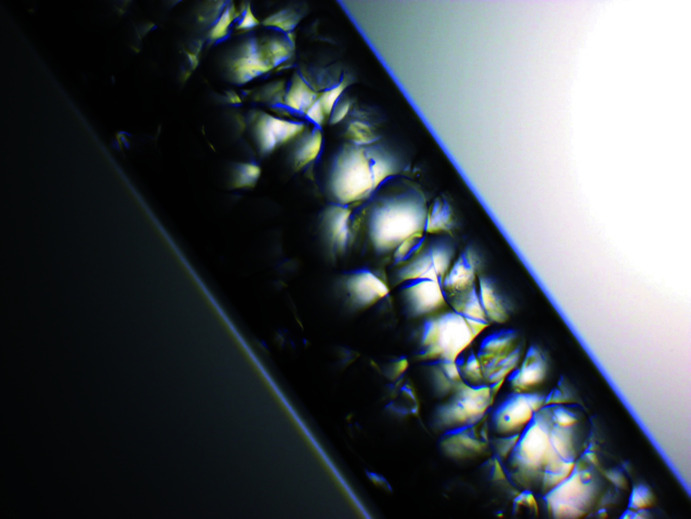
Image of a Hilgenberg capillary (diameter 0.5 mm) filled with crystals of LiNa_3_(SO_4_)_2_·6H_2_O by means of centrifugal compaction.

**Figure 15:**
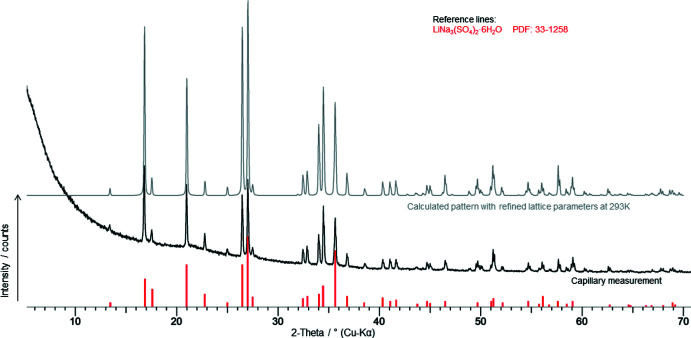
Powder XRD pattern of LiNa_3_(SO_4_)_2_·6H_2_O recorded from a rotating capillary. Scan rate: 20 sec, steps 0.023°. For comparison, the calculated powder pattern from structural data at 293 K is also shown.

Attempts were made to record powder XRD patterns from quickly ground crystals. Large crystals appear stable at least for some minutes on a filter paper. However, when grinding to achieve a crystal powder, dehydration took place. In cases of less intensive grinding, the texture effects were too large for a representative powder XRD pattern. Thus, particularly for powder XRD measurements, a suspension of fine crystals was prepared: To a 2 molar solution of Na_2_SO_4_, an equivalent amount of anhydrous Li_2_SO_4_ was added. The suspension was stirred two days at 298 K. The supernatant solution was deca­nted and subsequently some slurry was transferred into the expanded, upper part of a Hilgenberg glass capillary. By means of a centrifuge (30 minutes at 4000 r.p.m.), the crystals were pressed into the capillary. This way the available capillary volume was effectively filled with crystals (Fig. 14 ▸). A PXRD pattern obtained under rotation is shown in Fig. 15 ▸ in comparison with the one calculated from the crystal structure.

The powder pattern was measured at room temperature on a Bruker D8 Discover diffractometer in Bragg–Brentano geometry with Cu *K*α radiation (λ = 1.5406 Å) and a linear detector Våntec-1 (geometry angle 1°). The measurements were made with a Göbel mirror as monochromator with a 1.0 mm slit and a 2.5° primary soller. The generator was set to 40 kV/40 mA. The program *TOPAS 5.0* (Bruker, 2009 ▸) was used to refine the lattice parameters (Fig. 1 ▸). The solved structure from single crystal XRD at 293K was used as starting point of the refinement.

**Figure 16:**
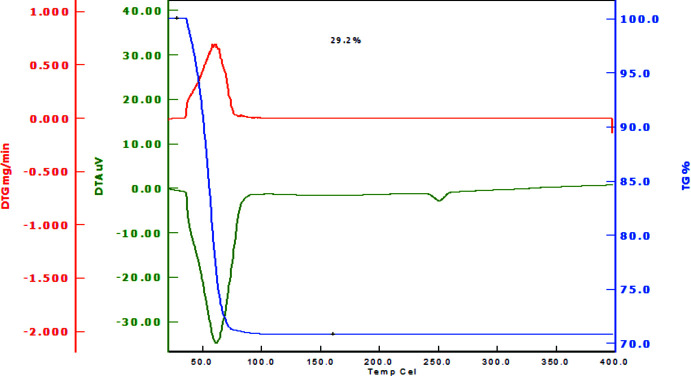
Thermal analysis of LiNa_3_(SO_4_)_2_·6H_2_O. Heating rate: 5 K min^−1^; N_2_ purge: 300 ml min^−1^.

Thermal analyses (Fig. 16 ▸) were performed from roughly crushed, large single crystals. Water is released in one step below 353 K. The mass loss of 29.2% is near the theoretical value of 28.7%. In a second experiment, the measured value was 29.1%.

## Refinement   

Crystal data, data collection and structure refinement details are summarized in Table 2 ▸. Structure solution using direct methods and a refinement of the atomic positions with respect to the isotropic displacement parameters led to the positions of the Na, Li, S and O atoms. The positions of the H atoms could be located from residual electron-density maxima after further refinement. H atoms were refined isotropically.

## Supplementary Material

Crystal structure: contains datablock(s) Na3_Li_2SO4_6H2O-90K, Na3_Li_2SO4_6H2O-180K, Na3_Li_2SO4_6H2O-260K, Na3_Li_2SO4_6H2O-273K, Na3_Li_2SO4_6H2O-293K, Na3_Li_2SO4_6H2O. DOI: 10.1107/S2056989021008057/ru2076sup1.cif

Structure factors: contains datablock(s) Na3_Li_2SO4_6H2O-90K. DOI: 10.1107/S2056989021008057/ru2076Na3_Li_2SO4_6H2O-90Ksup2.hkl

Structure factors: contains datablock(s) Na3_Li_2SO4_6H2O-180K. DOI: 10.1107/S2056989021008057/ru2076Na3_Li_2SO4_6H2O-180Ksup3.hkl

Structure factors: contains datablock(s) Na3_Li_2SO4_6H2O-260K. DOI: 10.1107/S2056989021008057/ru2076Na3_Li_2SO4_6H2O-260Ksup4.hkl

Structure factors: contains datablock(s) Na3_Li_2SO4_6H2O-273K. DOI: 10.1107/S2056989021008057/ru2076Na3_Li_2SO4_6H2O-273Ksup5.hkl

Structure factors: contains datablock(s) Na3_Li_2SO4_6H2O-293K. DOI: 10.1107/S2056989021008057/ru2076Na3_Li_2SO4_6H2O-293Ksup6.hkl

CCDC references: 2101588, 2101587, 2101586, 2101585, 2101584

Additional supporting information:  crystallographic information; 3D view; checkCIF report

## Figures and Tables

**Table 1:** Hydrogen-bond geometry (Å, °) at 273 K

*D*—H⋯*A*	*D*—H	H⋯*A*	*D*⋯*A*	*D*—H⋯*A*
O6—H6*A*⋯O2^i^	0.77 (5)	2.00 (5)	2.764 (11)	170 (5)
O6—H6*B*⋯O1	0.83 (4)	2.15 (4)	2.937 (7)	157 (4)
O5—H5*B*⋯O1^ii^	0.79 (4)	1.92 (4)	2.694 (7)	165 (4)
O5—H5*A*⋯O2^iii^	0.89 (4)	1.97 (4)	2.828 (6)	163 (3)

Symmetry codes: (i) -y+{\script{1\over 3}}, -x+{\script{2\over 3}}, z+{\script{1\over 6}}; (ii) x-{\script{1\over 3}}, x-y+{\script{1\over 3}}, z-{\script{1\over 6}}; (iii) -x+y, -x+1, z.

**Table 2:** Experimental details

	90 K	180 K	260 K	273 K	293 K
Crystal data					
Chemical formula	LiNa_3_(SO_4_)_2_·6H_2_O	LiNa_3_(SO_4_)_2_·6H_2_O	LiNa_3_(SO_4_)_2_·6H_2_O	LiNa_3_(SO_4_)_2_·6H_2_O	LiNa_3_(SO_4_)_2_·6H_2_O
*M*_r_	376.13	376.13	376.13	376.13	376.13
Crystal system, space group	Trigonal, *R*3*c*:*H*	Trigonal, *R*3*c*:*H*	Trigonal, *R*3*c*:*H*	Trigonal, *R*3*c*:*H*	Trigonal, *R*3*c*:*H*
*a*, *c* (Å)	8.3876 (13), 30.048 (7)	8.4006 (19), 30.111 (9)	8.426 (2), 30.197 (4)	8.4337 (17), 30.235 (6)	8.457 (7), 30.33 (3)
*V* (Å^3^)	1830.7 (7)	1840.3 (10)	1856.6 (10)	1862.4 (8)	1879 (4)
*Z*	6	6	6	6	6
Radiation type	Mo *K*α	Mo *K*α	Mo *K*α	Mo *K*α	Mo *K*α
μ (mm^−1^)	0.62	0.61	0.61	0.61	0.60
Crystal size (mm)	0.3 × 0.15 × 0.1	0.3 × 0.15 × 0.1	0.3 × 0.15 × 0.1	0.3 × 0.15 × 0.1	0.3 × 0.15 × 0.1

Data collection					
Diffractometer	Stoe IPDS 2	Stoe IPDS 2	Stoe IPDS 2	Stoe IPDS 2	Stoe IPDS 2T
Absorption correction	Integration Coppens (1970 ▸)	Integration Coppens (1970 ▸)	Integration Coppens (1970 ▸)	Integration Coppens (1970 ▸)	Integration Coppens (1970 ▸)
*T*_min_, *T*_max_	0.924, 0.945	0.682, 0.941	0.864, 0.938	0.773, 0.866	0.517, 0.844
No. of measured, independent and observed [*I* > 2σ(*I*)] reflections	12154, 1089, 1089	4345, 1088, 1087	5068, 915, 906	8584, 1159, 1141	1376, 730, 695
*R*_int_	0.027	0.035	0.034	0.035	0.054
(sin θ/λ)_max_ (Å^−1^)	0.685	0.692	0.643	0.694	0.641

Refinement					
*R*[*F*^2^ > 2σ(*F* ^2^)], *wR*(*F* ^2^), *S*	0.013, 0.036, 1.14	0.021, 0.053, 1.18	0.017, 0.043, 1.13	0.019, 0.053, 1.28	0.038, 0.105, 1.11
No. of reflections	1089	1088	915	1159	730
No. of parameters	78	78	78	77	78
No. of restraints	5	1	1	1	5
H-atom treatment	All H-atom parameters refined	All H-atom parameters refined	All H-atom parameters refined	All H-atom parameters refined	All H-atom parameters refined
Δρ_max_, Δρ_min_ (e Å^−3^)	0.16, −0.18	0.23, −0.33	0.14, −0.20	0.19, −0.33	0.33, −0.47
Absolute structure	Flack *x* determined using 539 quotients [(*I* ^+^)−(*I* ^−^)]/[(*I* ^+^)+(*I* ^−^)] (Parsons *et al.*, 2013 ▸)	Classical Flack method preferred over Parsons because s.u. lower	Flack *x* determined using 437 quotients [(*I* ^+^)−(*I* ^−^)]/[(*I* ^+^)+(*I* ^−^)] (Parsons *et al.*, 2013 ▸)	Flack *x* determined using 551 quotients [(*I* ^+^)−(*I* ^−^)]/[(*I* ^+^)+(*I* ^−^)] (Parsons *et al.*, 2013 ▸)	Classical Flack method preferred over Parsons because s.u. lower
Absolute structure parameter	0.01 (3)	−0.08 (10)	−0.04 (6)	0.04 (4)	−0.3 (3)

Computer programs: *X-AREA* and *X-RED* (Stoe & Cie, 2015 ▸), *SHELXS97* (Sheldrick, 2008 ▸), *SHELXL2018/3* (Sheldrick, 2015 ▸), *DIAMOND* (Brandenburg, 2017 ▸) and *publCIF* (Westrip, 2010 ▸).

## supplementary crystallographic information

### Lithium trisodium bis(sulfate) hexahydrate (Na3_Li_2SO4_6H2O-90K) Crystal data

**Table:**

LiNa_3_(SO_4_)_2_·6H_2_O	*D*_x_ = 2.047 Mg m^−^^3^
*M**_r_* = 376.13	Mo *K*α radiation, λ = 0.71073 Å
Trigonal, *R*3*c*:*H*	Cell parameters from 2749 reflections
*a* = 8.3876 (13) Å	θ = 25.0–27.5°
*c* = 30.048 (7) Å	µ = 0.62 mm^−^^1^
*V* = 1830.7 (7) Å^3^	*T* = 90 K
*Z* = 6	Needle, colourless
*F*(000) = 1152	0.3 × 0.15 × 0.1 mm

### Lithium trisodium bis(sulfate) hexahydrate (Na3_Li_2SO4_6H2O-90K) Data collection

**Table:**

Stoe IPDS 2 diffractometer	1089 independent reflections
Radiation source: sealed X-ray tube, 12 x 0.4 mm long-fine focus	1089 reflections with *I* > 2σ(*I*)
Plane graphite monochromator	*R*_int_ = 0.027
Detector resolution: 6.67 pixels mm^-1^	θ_max_ = 29.1°, θ_min_ = 3.1°
rotation method scans	*h* = −10→11
Absorption correction: integration Coppens (1970)	*k* = −11→11
*T*_min_ = 0.924, *T*_max_ = 0.945	*l* = −40→40
12154 measured reflections	

### Lithium trisodium bis(sulfate) hexahydrate (Na3_Li_2SO4_6H2O-90K) Refinement

**Table:**

Refinement on *F*^2^	All H-atom parameters refined
Least-squares matrix: full	*w* = 1/[σ^2^(*F*_o_^2^) + (0.0238*P*)^2^ + 0.6042*P*] where *P* = (*F*_o_^2^ + 2*F*_c_^2^)/3
*R*[*F*^2^ > 2σ(*F*^2^)] = 0.013	(Δ/σ)_max_ < 0.001
*wR*(*F*^2^) = 0.036	Δρ_max_ = 0.16 e Å^−^^3^
*S* = 1.14	Δρ_min_ = −0.18 e Å^−^^3^
1089 reflections	Extinction correction: SHELXL2018/3 (Sheldrick 2015), Fc^*^=kFc[1+0.001xFc^2^λ^3^/sin(2θ)]^-1/4^
78 parameters	Extinction coefficient: 0.0037 (7)
5 restraints	Absolute structure: Flack *x* determined using 539 quotients [(*I*^+^)-(*I*^-^)]/[(*I*^+^)+(*I*^-^)] (Parsons *et al.*, 2013)
Hydrogen site location: difference Fourier map	Absolute structure parameter: 0.01 (3)

### Lithium trisodium bis(sulfate) hexahydrate (Na3_Li_2SO4_6H2O-90K) Special details

**Table:**

Geometry. All esds (except the esd in the dihedral angle between two l.s. planes) are estimated using the full covariance matrix. The cell esds are taken into account individually in the estimation of esds in distances, angles and torsion angles; correlations between esds in cell parameters are only used when they are defined by crystal symmetry. An approximate (isotropic) treatment of cell esds is used for estimating esds involving l.s. planes.

### Lithium trisodium bis(sulfate) hexahydrate (Na3_Li_2SO4_6H2O-90K) Fractional atomic coordinates and isotropic or equivalent isotropic displacement parameters (Å^2^)

**Table:**

	*x*	*y*	*z*	*U*_iso_*/*U*_eq_	
Li1	0.666667	0.333333	0.15432 (13)	0.0085 (7)	
Na1	0.23426 (7)	0.26504 (7)	0.06063 (2)	0.00705 (14)	
S1	0.666667	0.333333	0.04323 (2)	0.00332 (12)	
S2	0.333333	0.666667	0.12552 (2)	0.00325 (12)	
O1	0.39254 (13)	0.53800 (12)	0.10892 (3)	0.00609 (18)	
O2	0.51766 (12)	0.36335 (12)	0.02615 (3)	0.00667 (18)	
O3	0.666667	0.333333	0.09154 (6)	0.0095 (4)	
O4	0.333333	0.666667	0.17470 (6)	0.0067 (3)	
O5	0.14513 (13)	0.42897 (12)	0.01016 (3)	0.00656 (17)	
H5A	0.053 (2)	0.429 (3)	0.0194 (7)	0.016 (5)*	
H5B	0.106 (3)	0.363 (3)	−0.0120 (6)	0.020 (5)*	
O6	−0.02932 (13)	0.20979 (13)	0.10180 (3)	0.00744 (19)	
H6B	−0.007 (3)	0.316 (2)	0.1019 (8)	0.017 (5)*	
H6A	−0.022 (3)	0.190 (3)	0.1279 (5)	0.017 (5)*	

### Lithium trisodium bis(sulfate) hexahydrate (Na3_Li_2SO4_6H2O-90K) Atomic displacement parameters (Å^2^)

**Table:**

	*U*^11^	*U*^22^	*U*^33^	*U*^12^	*U*^13^	*U*^23^
Li1	0.0089 (10)	0.0089 (10)	0.0078 (16)	0.0044 (5)	0.000	0.000
Na1	0.0065 (2)	0.0076 (2)	0.0072 (2)	0.00369 (19)	0.00175 (17)	0.00177 (18)
S1	0.00352 (15)	0.00352 (15)	0.0029 (2)	0.00176 (8)	0.000	0.000
S2	0.00334 (15)	0.00334 (15)	0.0031 (2)	0.00167 (8)	0.000	0.000
O1	0.0066 (4)	0.0060 (4)	0.0074 (4)	0.0045 (3)	−0.0002 (3)	−0.0015 (3)
O2	0.0057 (4)	0.0084 (4)	0.0079 (4)	0.0050 (3)	−0.0001 (3)	0.0013 (3)
O3	0.0129 (5)	0.0129 (5)	0.0028 (8)	0.0065 (3)	0.000	0.000
O4	0.0085 (5)	0.0085 (5)	0.0031 (7)	0.0042 (2)	0.000	0.000
O5	0.0063 (4)	0.0067 (4)	0.0068 (4)	0.0034 (3)	0.0010 (3)	−0.0010 (3)
O6	0.0089 (4)	0.0068 (4)	0.0064 (4)	0.0038 (4)	0.0000 (3)	0.0003 (3)

### Lithium trisodium bis(sulfate) hexahydrate (Na3_Li_2SO4_6H2O-90K) Geometric parameters (Å, º)

**Table:**

Li1—O3	1.886 (4)	Na1—O4^v^	2.6330 (14)
Li1—O5^i^	1.9434 (17)	Na1—Na1^vi^	3.6475 (10)
Li1—O5^ii^	1.9435 (17)	Na1—Na1^iv^	3.6475 (10)
Li1—O5^iii^	1.9435 (17)	S1—O3	1.4515 (17)
Li1—Na1^i^	3.748 (2)	S1—O2^vii^	1.4844 (9)
Li1—Na1^iii^	3.748 (2)	S1—O2	1.4844 (9)
Li1—Na1^ii^	3.748 (2)	S1—O2^viii^	1.4844 (9)
Na1—O2	2.3331 (10)	S2—O4	1.4777 (17)
Na1—O6^iv^	2.3498 (11)	S2—O1^ix^	1.4818 (9)
Na1—O6	2.3682 (11)	S2—O1	1.4818 (9)
Na1—O5	2.4036 (11)	S2—O1^x^	1.4818 (9)
Na1—O1	2.4638 (10)		
			
O3—Li1—O5^i^	110.37 (11)	O1—Na1—Na1^vi^	122.28 (3)
O3—Li1—O5^ii^	110.36 (11)	O4^v^—Na1—Na1^vi^	46.16 (3)
O5^i^—Li1—O5^ii^	108.56 (12)	O2—Na1—Na1^iv^	102.41 (3)
O3—Li1—O5^iii^	110.36 (11)	O6^iv^—Na1—Na1^iv^	39.55 (3)
O5^i^—Li1—O5^iii^	108.56 (12)	O6—Na1—Na1^iv^	85.42 (3)
O5^ii^—Li1—O5^iii^	108.56 (12)	O5—Na1—Na1^iv^	123.78 (3)
O3—Li1—Na1^i^	125.81 (5)	O1—Na1—Na1^iv^	142.40 (2)
O5^i^—Li1—Na1^i^	34.21 (6)	O4^v^—Na1—Na1^iv^	46.16 (3)
O5^ii^—Li1—Na1^i^	74.41 (8)	Na1^vi^—Na1—Na1^iv^	60.0
O5^iii^—Li1—Na1^i^	119.02 (15)	O2—Na1—Li1^xi^	72.17 (3)
O3—Li1—Na1^iii^	125.81 (5)	O6^iv^—Na1—Li1^xi^	167.14 (3)
O5^i^—Li1—Na1^iii^	74.40 (8)	O6—Na1—Li1^xi^	104.31 (4)
O5^ii^—Li1—Na1^iii^	119.01 (15)	O5—Na1—Li1^xi^	27.04 (3)
O5^iii^—Li1—Na1^iii^	34.21 (6)	O1—Na1—Li1^xi^	74.64 (5)
Na1^i^—Li1—Na1^iii^	89.23 (7)	O4^v^—Na1—Li1^xi^	98.29 (5)
O3—Li1—Na1^ii^	125.81 (5)	Na1^vi^—Na1—Li1^xi^	105.94 (2)
O5^i^—Li1—Na1^ii^	119.02 (15)	Na1^iv^—Na1—Li1^xi^	142.95 (5)
O5^ii^—Li1—Na1^ii^	34.21 (6)	O3—S1—O2^vii^	110.23 (4)
O5^iii^—Li1—Na1^ii^	74.40 (8)	O3—S1—O2	110.23 (4)
Na1^i^—Li1—Na1^ii^	89.23 (7)	O2^vii^—S1—O2	108.71 (5)
Na1^iii^—Li1—Na1^ii^	89.23 (7)	O3—S1—O2^viii^	110.23 (4)
O2—Na1—O6^iv^	95.05 (4)	O2^vii^—S1—O2^viii^	108.70 (5)
O2—Na1—O6	170.96 (4)	O2—S1—O2^viii^	108.71 (5)
O6^iv^—Na1—O6	88.14 (5)	O4—S2—O1^ix^	109.66 (4)
O2—Na1—O5	94.07 (4)	O4—S2—O1	109.66 (4)
O6^iv^—Na1—O5	162.69 (4)	O1^ix^—S2—O1	109.28 (4)
O6—Na1—O5	85.10 (4)	O4—S2—O1^x^	109.66 (4)
O2—Na1—O1	87.29 (4)	O1^ix^—S2—O1^x^	109.28 (4)
O6^iv^—Na1—O1	104.11 (4)	O1—S2—O1^x^	109.28 (4)
O6—Na1—O1	83.73 (3)	S2—O1—Na1	130.83 (5)
O5—Na1—O1	90.99 (3)	S1—O2—Na1	125.59 (6)
O2—Na1—O4^v^	103.31 (4)	S1—O3—Li1	180.0
O6^iv^—Na1—O4^v^	85.71 (4)	S2—O4—Na1^xii^	126.89 (4)
O6—Na1—O4^v^	85.34 (4)	S2—O4—Na1^xiii^	126.89 (4)
O5—Na1—O4^v^	77.88 (4)	Na1^xii^—O4—Na1^xiii^	87.68 (5)
O1—Na1—O4^v^	165.02 (3)	S2—O4—Na1^i^	126.89 (4)
O2—Na1—Na1^vi^	149.40 (3)	Na1^xii^—O4—Na1^i^	87.68 (5)
O6^iv^—Na1—Na1^vi^	85.68 (3)	Na1^xiii^—O4—Na1^i^	87.68 (5)
O6—Na1—Na1^vi^	39.18 (3)	Li1^xi^—O5—Na1	118.75 (8)
O5—Na1—Na1^vi^	79.01 (3)	Na1^vi^—O6—Na1	101.27 (4)

Symmetry codes: (i) *x*+1/3, *x*−*y*+2/3, *z*+1/6; (ii) −*y*+4/3, −*x*+2/3, *z*+1/6; (iii) −*x*+*y*+1/3, *y*−1/3, *z*+1/6; (iv) −*x*+*y*, −*x*, *z*; (v) −*y*+2/3, −*x*+1/3, *z*−1/6; (vi) −*y*, *x*−*y*, *z*; (vii) −*x*+*y*+1, −*x*+1, *z*; (viii) −*y*+1, *x*−*y*, *z*; (ix) −*y*+1, *x*−*y*+1, *z*; (x) −*x*+*y*, −*x*+1, *z*; (xi) −*y*+2/3, −*x*+4/3, *z*−1/6; (xii) −*x*+*y*+1/3, *y*+2/3, *z*+1/6; (xiii) −*y*+1/3, −*x*+2/3, *z*+1/6.

### Sodium-Lithium-Sulfate-Hexahydrate (Na3_Li_2SO4_6H2O-180K) Crystal data

**Table:**

3(Na)Li2(SO_4_)6(H2O)	*D*_x_ = 2.036 Mg m^−^^3^
*M**_r_* = 376.13	Mo *K*α radiation, λ = 0.71073 Å
Trigonal, *R*3*c*:*H*	Cell parameters from 7724 reflections
*a* = 8.4006 (19) Å	θ = 2.8–30.0°
*c* = 30.111 (9) Å	µ = 0.61 mm^−^^1^
*V* = 1840.3 (10) Å^3^	*T* = 180 K
*Z* = 6	Needle, colourless
*F*(000) = 1152	0.3 × 0.15 × 0.1 mm

### Sodium-Lithium-Sulfate-Hexahydrate (Na3_Li_2SO4_6H2O-180K) Data collection

**Table:**

Stoe IPDS 2 diffractometer	1088 independent reflections
Radiation source: sealed X-ray tube, 12 x 0.4 mm long-fine focus	1087 reflections with *I* > 2σ(*I*)
Plane graphite monochromator	*R*_int_ = 0.035
Detector resolution: 6.67 pixels mm^-1^	θ_max_ = 29.5°, θ_min_ = 3.1°
rotation method scans	*h* = −11→11
Absorption correction: integration Coppens (1970)	*k* = −11→11
*T*_min_ = 0.682, *T*_max_ = 0.941	*l* = −40→40
4345 measured reflections	

### Sodium-Lithium-Sulfate-Hexahydrate (Na3_Li_2SO4_6H2O-180K) Refinement

**Table:**

Refinement on *F*^2^	All H-atom parameters refined
Least-squares matrix: full	*w* = 1/[σ^2^(*F*_o_^2^) + (0.0413*P*)^2^] where *P* = (*F*_o_^2^ + 2*F*_c_^2^)/3
*R*[*F*^2^ > 2σ(*F*^2^)] = 0.021	(Δ/σ)_max_ < 0.001
*wR*(*F*^2^) = 0.053	Δρ_max_ = 0.23 e Å^−^^3^
*S* = 1.18	Δρ_min_ = −0.33 e Å^−^^3^
1088 reflections	Extinction correction: SHELXL2018/3 (Sheldrick 2015), Fc^*^=kFc[1+0.001xFc^2^λ^3^/sin(2θ)]^-1/4^
78 parameters	Extinction coefficient: 0.055 (4)
1 restraint	Absolute structure: Classical Flack method preferred over Parsons because s.u. lower
Hydrogen site location: difference Fourier map	Absolute structure parameter: −0.08 (10)

### Sodium-Lithium-Sulfate-Hexahydrate (Na3_Li_2SO4_6H2O-180K) Special details

**Table:**

Geometry. All esds (except the esd in the dihedral angle between two l.s. planes) are estimated using the full covariance matrix. The cell esds are taken into account individually in the estimation of esds in distances, angles and torsion angles; correlations between esds in cell parameters are only used when they are defined by crystal symmetry. An approximate (isotropic) treatment of cell esds is used for estimating esds involving l.s. planes.

### Sodium-Lithium-Sulfate-Hexahydrate (Na3_Li_2SO4_6H2O-180K) Fractional atomic coordinates and isotropic or equivalent isotropic displacement parameters (Å^2^)

**Table:**

	*x*	*y*	*z*	*U*_iso_*/*U*_eq_	
Li1	0.666667	0.333333	0.15397 (15)	0.0112 (7)	
Na1	0.23455 (8)	0.26585 (8)	0.06065 (3)	0.0123 (2)	
S1	0.666667	0.333333	0.04314 (2)	0.00567 (18)	
S2	0.333333	0.666667	0.12563 (2)	0.00580 (18)	
O1	0.39232 (16)	0.53835 (14)	0.10903 (4)	0.0101 (2)	
O2	0.51801 (14)	0.36299 (14)	0.02615 (4)	0.0116 (2)	
O3	0.666667	0.333333	0.09124 (10)	0.0182 (6)	
O4	0.333333	0.666667	0.17460 (9)	0.0113 (5)	
O5	0.14543 (15)	0.42931 (14)	0.00995 (4)	0.0107 (2)	
H5A	0.057 (4)	0.431 (3)	0.0205 (10)	0.019 (6)*	
H5B	0.106 (4)	0.359 (4)	−0.0106 (11)	0.021 (5)*	
O6	−0.02970 (15)	0.20921 (15)	0.10179 (4)	0.0125 (2)	
H6B	−0.011 (4)	0.311 (5)	0.1015 (12)	0.029 (8)*	
H6A	−0.023 (4)	0.189 (4)	0.1275 (11)	0.019 (6)*	

### Sodium-Lithium-Sulfate-Hexahydrate (Na3_Li_2SO4_6H2O-180K) Atomic displacement parameters (Å^2^)

**Table:**

	*U*^11^	*U*^22^	*U*^33^	*U*^12^	*U*^13^	*U*^23^
Li1	0.0125 (11)	0.0125 (11)	0.0087 (17)	0.0062 (5)	0.000	0.000
Na1	0.0118 (3)	0.0139 (3)	0.0119 (3)	0.0068 (2)	0.00347 (19)	0.00357 (19)
S1	0.0068 (2)	0.0068 (2)	0.0033 (3)	0.00342 (11)	0.000	0.000
S2	0.0064 (2)	0.0064 (2)	0.0046 (3)	0.00320 (11)	0.000	0.000
O1	0.0119 (4)	0.0099 (4)	0.0107 (5)	0.0073 (3)	−0.0002 (3)	−0.0020 (3)
O2	0.0095 (4)	0.0153 (5)	0.0131 (4)	0.0085 (4)	0.0002 (3)	0.0022 (3)
O3	0.0253 (9)	0.0253 (9)	0.0039 (11)	0.0127 (4)	0.000	0.000
O4	0.0143 (7)	0.0143 (7)	0.0054 (11)	0.0071 (4)	0.000	0.000
O5	0.0117 (4)	0.0099 (4)	0.0103 (5)	0.0051 (3)	0.0010 (4)	−0.0011 (3)
O6	0.0150 (5)	0.0122 (5)	0.0096 (5)	0.0064 (4)	0.0007 (3)	0.0000 (4)

### Sodium-Lithium-Sulfate-Hexahydrate (Na3_Li_2SO4_6H2O-180K) Geometric parameters (Å, º)

**Table:**

Li1—O3	1.889 (6)	Na1—O4^v^	2.644 (2)
Li1—O5^i^	1.9454 (19)	Na1—Na1^iv^	3.6617 (13)
Li1—O5^ii^	1.9455 (19)	Na1—Na1^vi^	3.6618 (13)
Li1—O5^iii^	1.9455 (19)	S1—O3	1.448 (3)
Li1—Na1^ii^	3.756 (3)	S1—O2	1.4813 (11)
Li1—Na1^iii^	3.756 (3)	S1—O2^vii^	1.4814 (11)
Li1—Na1^i^	3.756 (3)	S1—O2^viii^	1.4814 (11)
Na1—O2	2.3393 (12)	S2—O4	1.474 (3)
Na1—O6^iv^	2.3546 (13)	S2—O1	1.4804 (11)
Na1—O6	2.3734 (13)	S2—O1^ix^	1.4804 (11)
Na1—O5	2.4093 (13)	S2—O1^x^	1.4804 (11)
Na1—O1	2.4669 (12)		
			
O3—Li1—O5^i^	110.52 (13)	O1—Na1—Na1^iv^	142.29 (3)
O3—Li1—O5^ii^	110.52 (13)	O4^v^—Na1—Na1^iv^	46.17 (4)
O5^i^—Li1—O5^ii^	108.41 (13)	O2—Na1—Na1^vi^	149.23 (3)
O3—Li1—O5^iii^	110.52 (13)	O6^iv^—Na1—Na1^vi^	85.51 (3)
O5^i^—Li1—O5^iii^	108.41 (13)	O6—Na1—Na1^vi^	39.06 (3)
O5^ii^—Li1—O5^iii^	108.40 (13)	O5—Na1—Na1^vi^	79.08 (3)
O3—Li1—Na1^ii^	126.01 (6)	O1—Na1—Na1^vi^	122.25 (3)
O5^i^—Li1—Na1^ii^	118.70 (17)	O4^v^—Na1—Na1^vi^	46.17 (4)
O5^ii^—Li1—Na1^ii^	34.19 (6)	Na1^iv^—Na1—Na1^vi^	60.0
O5^iii^—Li1—Na1^ii^	74.26 (9)	O2—Na1—Li1^xi^	72.31 (3)
O3—Li1—Na1^iii^	126.01 (6)	O6^iv^—Na1—Li1^xi^	167.18 (4)
O5^i^—Li1—Na1^iii^	74.26 (9)	O6—Na1—Li1^xi^	104.59 (4)
O5^ii^—Li1—Na1^iii^	118.70 (17)	O5—Na1—Li1^xi^	26.99 (3)
O5^iii^—Li1—Na1^iii^	34.19 (6)	O1—Na1—Li1^xi^	74.93 (6)
Na1^ii^—Li1—Na1^iii^	88.94 (8)	O4^v^—Na1—Li1^xi^	98.14 (7)
O3—Li1—Na1^i^	126.01 (6)	Na1^iv^—Na1—Li1^xi^	142.78 (5)
O5^i^—Li1—Na1^i^	34.19 (6)	Na1^vi^—Na1—Li1^xi^	105.95 (2)
O5^ii^—Li1—Na1^i^	74.27 (9)	O3—S1—O2	110.20 (6)
O5^iii^—Li1—Na1^i^	118.70 (17)	O3—S1—O2^vii^	110.21 (6)
Na1^ii^—Li1—Na1^i^	88.94 (8)	O2—S1—O2^vii^	108.73 (6)
Na1^iii^—Li1—Na1^i^	88.94 (8)	O3—S1—O2^viii^	110.21 (6)
O2—Na1—O6^iv^	94.92 (4)	O2—S1—O2^viii^	108.73 (6)
O2—Na1—O6	171.37 (5)	O2^vii^—S1—O2^viii^	108.73 (6)
O6^iv^—Na1—O6	87.90 (6)	O4—S2—O1	109.74 (6)
O2—Na1—O5	94.13 (4)	O4—S2—O1^ix^	109.74 (6)
O6^iv^—Na1—O5	162.50 (5)	O1—S2—O1^ix^	109.20 (6)
O6—Na1—O5	85.35 (5)	O4—S2—O1^x^	109.74 (6)
O2—Na1—O1	87.57 (4)	O1—S2—O1^x^	109.20 (6)
O6^iv^—Na1—O1	104.10 (4)	O1^ix^—S2—O1^x^	109.20 (6)
O6—Na1—O1	83.82 (4)	S2—O1—Na1	130.92 (7)
O5—Na1—O1	91.21 (4)	S1—O2—Na1	125.78 (7)
O2—Na1—O4^v^	103.11 (6)	S1—O3—Li1	180.0
O6^iv^—Na1—O4^v^	85.59 (5)	S2—O4—Na1^xii^	126.90 (6)
O6—Na1—O4^v^	85.22 (5)	S2—O4—Na1^xiii^	126.90 (6)
O5—Na1—O4^v^	77.78 (5)	Na1^xii^—O4—Na1^xiii^	87.67 (9)
O1—Na1—O4^v^	165.06 (3)	S2—O4—Na1^i^	126.90 (6)
O2—Na1—Na1^iv^	102.10 (4)	Na1^xii^—O4—Na1^i^	87.67 (9)
O6^iv^—Na1—Na1^iv^	39.43 (3)	Na1^xiii^—O4—Na1^i^	87.67 (9)
O6—Na1—Na1^iv^	85.26 (3)	Li1^xi^—O5—Na1	118.83 (9)
O5—Na1—Na1^iv^	123.70 (3)	Na1^vi^—O6—Na1	101.51 (5)

Symmetry codes: (i) *x*+1/3, *x*−*y*+2/3, *z*+1/6; (ii) −*y*+4/3, −*x*+2/3, *z*+1/6; (iii) −*x*+*y*+1/3, *y*−1/3, *z*+1/6; (iv) −*x*+*y*, −*x*, *z*; (v) −*y*+2/3, −*x*+1/3, *z*−1/6; (vi) −*y*, *x*−*y*, *z*; (vii) −*y*+1, *x*−*y*, *z*; (viii) −*x*+*y*+1, −*x*+1, *z*; (ix) −*x*+*y*, −*x*+1, *z*; (x) −*y*+1, *x*−*y*+1, *z*; (xi) −*y*+2/3, −*x*+4/3, *z*−1/6; (xii) −*x*+*y*+1/3, *y*+2/3, *z*+1/6; (xiii) −*y*+1/3, −*x*+2/3, *z*+1/6.

### Sodium-Lithium-Sulfate-Hexahydrate (Na3_Li_2SO4_6H2O-260K) Crystal data

**Table:**

3(Na)Li2(SO_4_)6(H2O)	*D*_x_ = 2.018 Mg m^−^^3^
*M**_r_* = 376.13	Mo *K*α radiation, λ = 0.71073 Å
Trigonal, *R*3*c*:*H*	Cell parameters from 4304 reflections
*a* = 8.426 (2) Å	θ = 2.6–21.0°
*c* = 30.197 (4) Å	µ = 0.61 mm^−^^1^
*V* = 1856.6 (10) Å^3^	*T* = 260 K
*Z* = 6	Needle, colourless
*F*(000) = 1152	0.3 × 0.15 × 0.1 mm

### Sodium-Lithium-Sulfate-Hexahydrate (Na3_Li_2SO4_6H2O-260K) Data collection

**Table:**

Stoe IPDS 2 diffractometer	915 independent reflections
Radiation source: sealed X-ray tube, 12 x 0.4 mm long-fine focus	906 reflections with *I* > 2σ(*I*)
Plane graphite monochromator	*R*_int_ = 0.034
Detector resolution: 6.67 pixels mm^-1^	θ_max_ = 27.2°, θ_min_ = 3.1°
rotation method scans	*h* = −10→10
Absorption correction: integration Coppens (1970)	*k* = −9→10
*T*_min_ = 0.864, *T*_max_ = 0.938	*l* = −38→38
5068 measured reflections	

### Sodium-Lithium-Sulfate-Hexahydrate (Na3_Li_2SO4_6H2O-260K) Refinement

**Table:**

Refinement on *F*^2^	All H-atom parameters refined
Least-squares matrix: full	*w* = 1/[σ^2^(*F*_o_^2^) + (0.0254*P*)^2^ + 0.5038*P*] where *P* = (*F*_o_^2^ + 2*F*_c_^2^)/3
*R*[*F*^2^ > 2σ(*F*^2^)] = 0.017	(Δ/σ)_max_ < 0.001
*wR*(*F*^2^) = 0.043	Δρ_max_ = 0.14 e Å^−^^3^
*S* = 1.13	Δρ_min_ = −0.20 e Å^−^^3^
915 reflections	Extinction correction: SHELXL2018/3 (Sheldrick 2015), Fc^*^=kFc[1+0.001xFc^2^λ^3^/sin(2θ)]^-1/4^
78 parameters	Extinction coefficient: 0.0031 (4)
1 restraint	Absolute structure: Flack *x* determined using 437 quotients [(*I*^+^)-(*I*^-^)]/[(*I*^+^)+(*I*^-^)] (Parsons *et al.*, 2013)
Hydrogen site location: difference Fourier map	Absolute structure parameter: −0.04 (6)

### Sodium-Lithium-Sulfate-Hexahydrate (Na3_Li_2SO4_6H2O-260K) Special details

**Table:**

Geometry. All esds (except the esd in the dihedral angle between two l.s. planes) are estimated using the full covariance matrix. The cell esds are taken into account individually in the estimation of esds in distances, angles and torsion angles; correlations between esds in cell parameters are only used when they are defined by crystal symmetry. An approximate (isotropic) treatment of cell esds is used for estimating esds involving l.s. planes.

### Sodium-Lithium-Sulfate-Hexahydrate (Na3_Li_2SO4_6H2O-260K) Fractional atomic coordinates and isotropic or equivalent isotropic displacement parameters (Å^2^)

**Table:**

	*x*	*y*	*z*	*U*_iso_*/*U*_eq_	
Li1	0.333333	0.666667	0.8465 (2)	0.0164 (12)	
Na1	0.76508 (11)	0.73321 (11)	0.93934 (4)	0.0204 (2)	
S1	0.333333	0.666667	0.95693 (2)	0.01085 (19)	
S2	0.666667	0.333333	0.87429 (2)	0.01117 (19)	
O1	0.6085 (2)	0.46143 (19)	0.89076 (5)	0.0167 (3)	
O2	0.4815 (2)	0.6374 (2)	0.97377 (5)	0.0188 (3)	
O3	0.333333	0.666667	0.90900 (9)	0.0282 (7)	
O4	0.666667	0.333333	0.82539 (8)	0.0181 (6)	
O5	0.8541 (2)	0.56971 (19)	0.99018 (5)	0.0172 (3)	
H5A	0.949 (5)	0.569 (4)	0.9803 (10)	0.027 (8)*	
H5B	0.890 (4)	0.630 (4)	1.0103 (11)	0.027 (8)*	
O6	1.0306 (2)	0.7917 (2)	0.89835 (6)	0.0200 (3)	
H6B	1.014 (5)	0.690 (6)	0.8961 (11)	0.045 (10)*	
H6A	1.017 (5)	0.807 (5)	0.8738 (12)	0.044 (10)*	

### Sodium-Lithium-Sulfate-Hexahydrate (Na3_Li_2SO4_6H2O-260K) Atomic displacement parameters (Å^2^)

**Table:**

	*U*^11^	*U*^22^	*U*^33^	*U*^12^	*U*^13^	*U*^23^
Li1	0.0178 (17)	0.0178 (17)	0.014 (3)	0.0089 (9)	0.000	0.000
Na1	0.0190 (4)	0.0218 (4)	0.0207 (4)	0.0104 (4)	0.0049 (3)	0.0054 (3)
S1	0.0115 (2)	0.0115 (2)	0.0096 (4)	0.00575 (12)	0.000	0.000
S2	0.0113 (2)	0.0113 (2)	0.0109 (4)	0.00565 (12)	0.000	0.000
O1	0.0181 (7)	0.0165 (7)	0.0194 (7)	0.0114 (6)	−0.0007 (5)	−0.0030 (5)
O2	0.0160 (7)	0.0236 (7)	0.0222 (7)	0.0138 (6)	0.0009 (6)	0.0032 (6)
O3	0.0384 (11)	0.0384 (11)	0.0076 (14)	0.0192 (6)	0.000	0.000
O4	0.0221 (9)	0.0221 (9)	0.0102 (12)	0.0110 (4)	0.000	0.000
O5	0.0184 (7)	0.0154 (7)	0.0171 (7)	0.0079 (6)	0.0020 (6)	−0.0013 (6)
O6	0.0223 (8)	0.0196 (8)	0.0179 (7)	0.0102 (7)	0.0005 (6)	−0.0009 (6)

### Sodium-Lithium-Sulfate-Hexahydrate (Na3_Li_2SO4_6H2O-260K) Geometric parameters (Å, º)

**Table:**

Li1—O3	1.888 (6)	Na1—O4^v^	2.656 (2)
Li1—O5^i^	1.948 (3)	Na1—Na1^iv^	3.6830 (17)
Li1—O5^ii^	1.948 (3)	Na1—Na1^vi^	3.6830 (17)
Li1—O5^iii^	1.948 (3)	S1—O3	1.447 (3)
Li1—Na1^iii^	3.770 (4)	S1—O2	1.4788 (15)
Li1—Na1^i^	3.770 (4)	S1—O2^vii^	1.4788 (15)
Li1—Na1^ii^	3.770 (4)	S1—O2^viii^	1.4788 (15)
Na1—O2	2.3478 (17)	S2—O4	1.477 (2)
Na1—O6^iv^	2.3584 (18)	S2—O1^ix^	1.4769 (14)
Na1—O6	2.3825 (19)	S2—O1^x^	1.4769 (14)
Na1—O5	2.4188 (16)	S2—O1	1.4769 (14)
Na1—O1	2.4727 (17)		
			
O3—Li1—O5^i^	110.85 (17)	O1—Na1—Na1^iv^	142.14 (4)
O3—Li1—O5^ii^	110.85 (17)	O4^v^—Na1—Na1^iv^	46.11 (4)
O5^i^—Li1—O5^ii^	108.06 (18)	O2—Na1—Na1^vi^	149.08 (5)
O3—Li1—O5^iii^	110.85 (17)	O6^iv^—Na1—Na1^vi^	85.36 (5)
O5^i^—Li1—O5^iii^	108.06 (18)	O6—Na1—Na1^vi^	38.79 (4)
O5^ii^—Li1—O5^iii^	108.06 (18)	O5—Na1—Na1^vi^	79.18 (5)
O3—Li1—Na1^iii^	126.24 (8)	O1—Na1—Na1^vi^	122.10 (5)
O5^i^—Li1—Na1^iii^	118.2 (2)	O4^v^—Na1—Na1^vi^	46.11 (4)
O5^ii^—Li1—Na1^iii^	73.96 (12)	Na1^iv^—Na1—Na1^vi^	60.000 (1)
O5^iii^—Li1—Na1^iii^	34.14 (9)	O2—Na1—Li1^xi^	72.52 (5)
O3—Li1—Na1^i^	126.24 (8)	O6^iv^—Na1—Li1^xi^	167.16 (6)
O5^i^—Li1—Na1^i^	34.14 (9)	O6—Na1—Li1^xi^	104.84 (6)
O5^ii^—Li1—Na1^i^	118.2 (2)	O5—Na1—Li1^xi^	26.88 (5)
O5^iii^—Li1—Na1^i^	73.96 (12)	O1—Na1—Li1^xi^	75.28 (8)
Na1^iii^—Li1—Na1^i^	88.61 (11)	O4^v^—Na1—Li1^xi^	98.04 (8)
O3—Li1—Na1^ii^	126.24 (8)	Na1^iv^—Na1—Li1^xi^	142.58 (7)
O5^i^—Li1—Na1^ii^	73.96 (12)	Na1^vi^—Na1—Li1^xi^	105.97 (3)
O5^ii^—Li1—Na1^ii^	34.14 (9)	O3—S1—O2	110.11 (7)
O5^iii^—Li1—Na1^ii^	118.2 (2)	O3—S1—O2^vii^	110.12 (7)
Na1^iii^—Li1—Na1^ii^	88.61 (11)	O2—S1—O2^vii^	108.82 (7)
Na1^i^—Li1—Na1^ii^	88.61 (11)	O3—S1—O2^viii^	110.12 (7)
O2—Na1—O6^iv^	94.66 (6)	O2—S1—O2^viii^	108.82 (7)
O2—Na1—O6	171.88 (7)	O2^vii^—S1—O2^viii^	108.82 (7)
O6^iv^—Na1—O6	87.76 (9)	O4—S2—O1^ix^	109.68 (7)
O2—Na1—O5	94.30 (6)	O4—S2—O1^x^	109.68 (7)
O6^iv^—Na1—O5	162.39 (7)	O1^ix^—S2—O1^x^	109.26 (7)
O6—Na1—O5	85.48 (7)	O4—S2—O1	109.69 (6)
O2—Na1—O1	87.96 (6)	O1^ix^—S2—O1	109.26 (7)
O6^iv^—Na1—O1	104.13 (6)	O1^x^—S2—O1	109.26 (7)
O6—Na1—O1	83.93 (6)	S2—O1—Na1	131.17 (9)
O5—Na1—O1	91.31 (6)	S1—O2—Na1	126.11 (9)
O2—Na1—O4^v^	103.01 (6)	S1—O3—Li1	180.0
O6^iv^—Na1—O4^v^	85.37 (6)	S2—O4—Na1^xii^	126.82 (5)
O6—Na1—O4^v^	84.90 (6)	S2—O4—Na1^xiii^	126.82 (5)
O5—Na1—O4^v^	77.85 (6)	Na1^xii^—O4—Na1^xiii^	87.78 (7)
O1—Na1—O4^v^	165.00 (5)	S2—O4—Na1^iii^	126.82 (5)
O2—Na1—Na1^iv^	101.74 (5)	Na1^xii^—O4—Na1^iii^	87.78 (7)
O6^iv^—Na1—Na1^iv^	39.26 (5)	Na1^xiii^—O4—Na1^iii^	87.78 (7)
O6—Na1—Na1^iv^	85.03 (4)	Li1^xi^—O5—Na1	118.98 (12)
O5—Na1—Na1^iv^	123.73 (5)	Na1^vi^—O6—Na1	101.95 (7)

Symmetry codes: (i) −*x*+*y*+2/3, *y*+1/3, *z*−1/6; (ii) −*y*+2/3, −*x*+4/3, *z*−1/6; (iii) *x*−1/3, *x*−*y*+1/3, *z*−1/6; (iv) −*x*+*y*+1, −*x*+2, *z*; (v) −*y*+4/3, −*x*+5/3, *z*+1/6; (vi) −*y*+2, *x*−*y*+1, *z*; (vii) −*y*+1, *x*−*y*+1, *z*; (viii) −*x*+*y*, −*x*+1, *z*; (ix) −*x*+*y*+1, −*x*+1, *z*; (x) −*y*+1, *x*−*y*, *z*; (xi) −*y*+4/3, −*x*+2/3, *z*+1/6; (xii) −*x*+*y*+2/3, *y*−2/3, *z*−1/6; (xiii) −*y*+5/3, −*x*+4/3, *z*−1/6.

### Sodium-Lithium-Sulfate-Hexahydrate (Na3_Li_2SO4_6H2O-273K) Crystal data

**Table:**

3(Na)Li2(SO_4_)6(H2O)	*D*_x_ = 2.012 Mg m^−^^3^
*M**_r_* = 376.13	Mo *K*α radiation, λ = 0.71073 Å
Trigonal, *R*3*c*:*H*	Cell parameters from 10790 reflections
*a* = 8.4337 (17) Å	θ = 2.8–27.5°
*c* = 30.235 (6) Å	µ = 0.61 mm^−^^1^
*V* = 1862.4 (8) Å^3^	*T* = 273 K
*Z* = 6	Needle, colourless
*F*(000) = 1152	0.3 × 0.15 × 0.1 mm

### Sodium-Lithium-Sulfate-Hexahydrate (Na3_Li_2SO4_6H2O-273K) Data collection

**Table:**

Stoe IPDS 2 diffractometer	1159 independent reflections
Radiation source: sealed X-ray tube, 12 x 0.4 mm long-fine focus	1141 reflections with *I* > 2σ(*I*)
Plane graphite monochromator	*R*_int_ = 0.035
Detector resolution: 6.67 pixels mm^-1^	θ_max_ = 29.6°, θ_min_ = 3.1°
rotation method scans	*h* = −11→11
Absorption correction: integration Coppens (1970)	*k* = −10→11
*T*_min_ = 0.773, *T*_max_ = 0.866	*l* = −40→40
8584 measured reflections	

### Sodium-Lithium-Sulfate-Hexahydrate (Na3_Li_2SO4_6H2O-273K) Refinement

**Table:**

Refinement on *F*^2^	Hydrogen site location: difference Fourier map
Least-squares matrix: full	All H-atom parameters refined
*R*[*F*^2^ > 2σ(*F*^2^)] = 0.019	*w* = 1/[σ^2^(*F*_o_^2^) + (0.0212*P*)^2^ + 1.6465*P*] where *P* = (*F*_o_^2^ + 2*F*_c_^2^)/3
*wR*(*F*^2^) = 0.053	(Δ/σ)_max_ < 0.001
*S* = 1.28	Δρ_max_ = 0.19 e Å^−^^3^
1159 reflections	Δρ_min_ = −0.33 e Å^−^^3^
77 parameters	Absolute structure: Flack *x* determined using 551 quotients [(*I*^+^)-(*I*^-^)]/[(*I*^+^)+(*I*^-^)] (Parsons *et al.*, 2013)
1 restraint	Absolute structure parameter: 0.04 (4)

### Sodium-Lithium-Sulfate-Hexahydrate (Na3_Li_2SO4_6H2O-273K) Special details

**Table:**

Geometry. All esds (except the esd in the dihedral angle between two l.s. planes) are estimated using the full covariance matrix. The cell esds are taken into account individually in the estimation of esds in distances, angles and torsion angles; correlations between esds in cell parameters are only used when they are defined by crystal symmetry. An approximate (isotropic) treatment of cell esds is used for estimating esds involving l.s. planes.

### Sodium-Lithium-Sulfate-Hexahydrate (Na3_Li_2SO4_6H2O-273K) Fractional atomic coordinates and isotropic or equivalent isotropic displacement parameters (Å^2^)

**Table:**

	*x*	*y*	*z*	*U*_iso_*/*U*_eq_	
Li1	0.666667	0.333333	0.1535 (2)	0.0180 (12)	
Na1	0.23496 (12)	0.26693 (13)	0.06069 (4)	0.0207 (2)	
S1	0.666667	0.333333	0.04304 (3)	0.01061 (16)	
S2	0.333333	0.666667	0.12574 (3)	0.01110 (16)	
O1	0.3914 (2)	0.5385 (2)	0.10929 (5)	0.0170 (3)	
O2	0.5185 (2)	0.3624 (2)	0.02624 (6)	0.0193 (3)	
O3	0.666667	0.333333	0.09098 (10)	0.0290 (7)	
O4	0.333333	0.666667	0.17459 (9)	0.0190 (5)	
O5	0.1459 (2)	0.4305 (2)	0.00968 (6)	0.0174 (3)	
H5A	0.047 (6)	0.434 (5)	0.0201 (13)	0.034 (10)*	
H5B	0.108 (5)	0.365 (5)	−0.0120 (13)	0.030 (9)*	
O6	−0.0306 (2)	0.2082 (2)	0.10174 (6)	0.0205 (3)	
H6B	−0.009 (6)	0.314 (7)	0.1031 (13)	0.043 (11)*	
H6A	−0.023 (7)	0.188 (6)	0.1279 (15)	0.051 (12)*	

### Sodium-Lithium-Sulfate-Hexahydrate (Na3_Li_2SO4_6H2O-273K) Atomic displacement parameters (Å^2^)

**Table:**

	*U*^11^	*U*^22^	*U*^33^	*U*^12^	*U*^13^	*U*^23^
Li1	0.0190 (18)	0.0190 (18)	0.016 (3)	0.0095 (9)	0.000	0.000
Na1	0.0189 (4)	0.0228 (5)	0.0206 (4)	0.0107 (4)	0.0053 (3)	0.0055 (4)
S1	0.0116 (2)	0.0116 (2)	0.0087 (3)	0.00578 (11)	0.000	0.000
S2	0.0112 (2)	0.0112 (2)	0.0108 (4)	0.00562 (11)	0.000	0.000
O1	0.0181 (7)	0.0173 (7)	0.0192 (7)	0.0117 (6)	−0.0011 (6)	−0.0032 (6)
O2	0.0166 (7)	0.0242 (8)	0.0224 (7)	0.0141 (6)	0.0000 (6)	0.0033 (6)
O3	0.0393 (12)	0.0393 (12)	0.0083 (13)	0.0196 (6)	0.000	0.000
O4	0.0230 (8)	0.0230 (8)	0.0110 (12)	0.0115 (4)	0.000	0.000
O5	0.0186 (7)	0.0158 (7)	0.0173 (7)	0.0083 (6)	0.0021 (6)	−0.0009 (6)
O6	0.0235 (9)	0.0193 (8)	0.0186 (8)	0.0106 (7)	0.0004 (6)	−0.0009 (7)

### Sodium-Lithium-Sulfate-Hexahydrate (Na3_Li_2SO4_6H2O-273K) Geometric parameters (Å, º)

**Table:**

Li1—O3	1.889 (7)	Na1—O4^v^	2.661 (2)
Li1—O5^i^	1.948 (3)	Na1—Na1^vi^	3.6879 (18)
Li1—O5^ii^	1.948 (3)	Na1—Na1^iv^	3.6879 (18)
Li1—O5^iii^	1.948 (3)	S1—O3	1.449 (3)
Li1—Na1^i^	3.775 (4)	S1—O2^vii^	1.4783 (16)
Li1—Na1^iii^	3.775 (4)	S1—O2	1.4783 (16)
Li1—Na1^ii^	3.775 (4)	S1—O2^viii^	1.4783 (16)
Na1—O2	2.3509 (19)	S2—O4	1.477 (3)
Na1—O6^iv^	2.362 (2)	S2—O1^ix^	1.4781 (16)
Na1—O6	2.386 (2)	S2—O1^x^	1.4781 (16)
Na1—O5	2.4253 (18)	S2—O1	1.4781 (16)
Na1—O1	2.4745 (18)		
			
O3—Li1—O5^i^	110.80 (19)	O1—Na1—Na1^vi^	122.09 (5)
O3—Li1—O5^ii^	110.80 (19)	O4^v^—Na1—Na1^vi^	46.13 (4)
O5^i^—Li1—O5^ii^	108.1 (2)	O2—Na1—Na1^iv^	101.65 (6)
O3—Li1—O5^iii^	110.80 (19)	O6^iv^—Na1—Na1^iv^	39.26 (5)
O5^i^—Li1—O5^iii^	108.1 (2)	O6—Na1—Na1^iv^	84.99 (5)
O5^ii^—Li1—O5^iii^	108.1 (2)	O5—Na1—Na1^iv^	123.70 (5)
O3—Li1—Na1^i^	126.29 (8)	O1—Na1—Na1^iv^	142.09 (4)
O5^i^—Li1—Na1^i^	34.22 (10)	O4^v^—Na1—Na1^iv^	46.13 (4)
O5^ii^—Li1—Na1^i^	73.93 (13)	Na1^vi^—Na1—Na1^iv^	60.0
O5^iii^—Li1—Na1^i^	118.2 (3)	O2—Na1—Li1^xi^	72.56 (5)
O3—Li1—Na1^iii^	126.29 (8)	O6^iv^—Na1—Li1^xi^	167.19 (6)
O5^i^—Li1—Na1^iii^	73.93 (13)	O6—Na1—Li1^xi^	104.94 (7)
O5^ii^—Li1—Na1^iii^	118.2 (3)	O5—Na1—Li1^xi^	26.85 (5)
O5^iii^—Li1—Na1^iii^	34.22 (10)	O1—Na1—Li1^xi^	75.37 (9)
Na1^i^—Li1—Na1^iii^	88.54 (12)	O4^v^—Na1—Li1^xi^	97.99 (9)
O3—Li1—Na1^ii^	126.29 (8)	Na1^vi^—Na1—Li1^xi^	105.97 (4)
O5^i^—Li1—Na1^ii^	118.2 (3)	Na1^iv^—Na1—Li1^xi^	142.53 (8)
O5^ii^—Li1—Na1^ii^	34.22 (10)	O3—S1—O2^vii^	110.10 (8)
O5^iii^—Li1—Na1^ii^	73.93 (13)	O3—S1—O2	110.10 (8)
Na1^i^—Li1—Na1^ii^	88.54 (12)	O2^vii^—S1—O2	108.83 (8)
Na1^iii^—Li1—Na1^ii^	88.54 (12)	O3—S1—O2^viii^	110.10 (8)
O2—Na1—O6^iv^	94.65 (7)	O2^vii^—S1—O2^viii^	108.83 (8)
O2—Na1—O6	171.96 (7)	O2—S1—O2^viii^	108.83 (8)
O6^iv^—Na1—O6	87.63 (9)	O4—S2—O1^ix^	109.66 (7)
O2—Na1—O5	94.28 (6)	O4—S2—O1^x^	109.66 (7)
O6^iv^—Na1—O5	162.35 (7)	O1^ix^—S2—O1^x^	109.28 (7)
O6—Na1—O5	85.62 (7)	O4—S2—O1	109.66 (7)
O2—Na1—O1	88.05 (7)	O1^ix^—S2—O1	109.28 (7)
O6^iv^—Na1—O1	104.07 (6)	O1^x^—S2—O1	109.28 (7)
O6—Na1—O1	83.92 (6)	S2—O1—Na1	131.18 (9)
O5—Na1—O1	91.41 (6)	S1—O2—Na1	126.18 (10)
O2—Na1—O4^v^	102.93 (7)	S1—O3—Li1	180.0
O6^iv^—Na1—O4^v^	85.39 (6)	S2—O4—Na1^xii^	126.84 (6)
O6—Na1—O4^v^	84.92 (6)	S2—O4—Na1^xiii^	126.84 (6)
O5—Na1—O4^v^	77.80 (6)	Na1^xii^—O4—Na1^xiii^	87.75 (8)
O1—Na1—O4^v^	165.02 (5)	S2—O4—Na1^i^	126.84 (6)
O2—Na1—Na1^vi^	149.02 (5)	Na1^xii^—O4—Na1^i^	87.75 (8)
O6^iv^—Na1—Na1^vi^	85.31 (5)	Na1^xiii^—O4—Na1^i^	87.75 (8)
O6—Na1—Na1^vi^	38.80 (5)	Li1^xi^—O5—Na1	118.93 (13)
O5—Na1—Na1^vi^	79.22 (5)	Na1^vi^—O6—Na1	101.94 (8)

Symmetry codes: (i) *x*+1/3, *x*−*y*+2/3, *z*+1/6; (ii) −*y*+4/3, −*x*+2/3, *z*+1/6; (iii) −*x*+*y*+1/3, *y*−1/3, *z*+1/6; (iv) −*x*+*y*, −*x*, *z*; (v) −*y*+2/3, −*x*+1/3, *z*−1/6; (vi) −*y*, *x*−*y*, *z*; (vii) −*y*+1, *x*−*y*, *z*; (viii) −*x*+*y*+1, −*x*+1, *z*; (ix) −*x*+*y*, −*x*+1, *z*; (x) −*y*+1, *x*−*y*+1, *z*; (xi) −*y*+2/3, −*x*+4/3, *z*−1/6; (xii) −*x*+*y*+1/3, *y*+2/3, *z*+1/6; (xiii) −*y*+1/3, −*x*+2/3, *z*+1/6.

### Sodium-Lithium-Sulfate-Hexahydrate (Na3_Li_2SO4_6H2O-273K) Hydrogen-bond geometry (Å, º)

**Table:**

*D*—H···*A*	*D*—H	H···*A*	*D*···*A*	*D*—H···*A*
O6—H6*A*···O2^xiii^	0.77 (5)	2.00 (5)	2.764 (11)	170 (5)
O6—H6*B*···O1	0.83 (4)	2.15 (4)	2.937 (7)	157 (4)
O5—H5*B*···O1^xiv^	0.79 (4)	1.92 (4)	2.694 (7)	165 (4)
O5—H5*A*···O2^ix^	0.89 (4)	1.97 (4)	2.828 (6)	163 (3)

Symmetry codes: (ix) −*x*+*y*, −*x*+1, *z*; (xiii) −*y*+1/3, −*x*+2/3, *z*+1/6; (xiv) *x*−1/3, *x*−*y*+1/3, *z*−1/6.

### (Na3_Li_2SO4_6H2O-293K) Crystal data

**Table:**

H_12_LiNa_3_O_14_S_2_	*D*_x_ = 1.995 Mg m^−^^3^
*M**_r_* = 376.13	Mo *K*α radiation, λ = 0.71073 Å
Trigonal, *R*3*c*:*H*	Cell parameters from 5111 reflections
*a* = 8.457 (7) Å	θ = 2.9–27.1°
*c* = 30.33 (3) Å	µ = 0.60 mm^−^^1^
*V* = 1879 (4) Å^3^	*T* = 293 K
*Z* = 6	Needle, colourless
*F*(000) = 1152	0.3 × 0.15 × 0.1 mm

### (Na3_Li_2SO4_6H2O-293K) Data collection

**Table:**

Stoe IPDS 2T diffractometer	730 independent reflections
Radiation source: sealed X-ray tube, 12 x 0.4 mm long-fine focus	695 reflections with *I* > 2σ(*I*)
Plane graphite monochromator	*R*_int_ = 0.054
Detector resolution: 6.67 pixels mm^-1^	θ_max_ = 27.1°, θ_min_ = 3.9°
rotation method scans	*h* = −9→10
Absorption correction: integration Coppens (1970)	*k* = −6→10
*T*_min_ = 0.517, *T*_max_ = 0.844	*l* = −38→35
1376 measured reflections	

### (Na3_Li_2SO4_6H2O-293K) Refinement

**Table:**

Refinement on *F*^2^	All H-atom parameters refined
Least-squares matrix: full	*w* = 1/[σ^2^(*F*_o_^2^) + (0.0771*P*)^2^ + 1.3675*P*] where *P* = (*F*_o_^2^ + 2*F*_c_^2^)/3
*R*[*F*^2^ > 2σ(*F*^2^)] = 0.038	(Δ/σ)_max_ < 0.001
*wR*(*F*^2^) = 0.105	Δρ_max_ = 0.33 e Å^−^^3^
*S* = 1.11	Δρ_min_ = −0.47 e Å^−^^3^
730 reflections	Extinction correction: SHELXL2018/3 (Sheldrick 2015), Fc^*^=kFc[1+0.001xFc^2^λ^3^/sin(2θ)]^-1/4^
78 parameters	Extinction coefficient: 0.0054 (14)
5 restraints	Absolute structure: Classical Flack method preferred over Parsons because s.u. lower
Hydrogen site location: difference Fourier map	Absolute structure parameter: −0.3 (3)

### (Na3_Li_2SO4_6H2O-293K) Special details

**Table:**

Geometry. All esds (except the esd in the dihedral angle between two l.s. planes) are estimated using the full covariance matrix. The cell esds are taken into account individually in the estimation of esds in distances, angles and torsion angles; correlations between esds in cell parameters are only used when they are defined by crystal symmetry. An approximate (isotropic) treatment of cell esds is used for estimating esds involving l.s. planes.

### (Na3_Li_2SO4_6H2O-293K) Fractional atomic coordinates and isotropic or equivalent isotropic displacement parameters (Å^2^)

**Table:**

	*x*	*y*	*z*	*U*_iso_*/*U*_eq_	
Li1	0.666667	0.333333	0.1539 (4)	0.026 (3)	
Na1	0.2353 (3)	0.2672 (3)	0.06061 (9)	0.0300 (5)	
S1	0.666667	0.333333	0.04301 (5)	0.0167 (4)	
S2	0.333333	0.666667	0.12572 (5)	0.0169 (4)	
O1	0.3912 (5)	0.5387 (4)	0.10917 (11)	0.0263 (7)	
O2	0.5187 (4)	0.3622 (5)	0.02643 (11)	0.0291 (7)	
O3	0.666667	0.333333	0.0906 (2)	0.0365 (18)	
O4	0.333333	0.666667	0.1745 (2)	0.0250 (13)	
O5	0.1454 (5)	0.4298 (4)	0.00960 (11)	0.0256 (7)	
H5A	0.059 (6)	0.440 (9)	0.018 (2)	0.035 (17)*	
H5B	0.096 (9)	0.354 (8)	−0.0099 (18)	0.039 (16)*	
O6	−0.0302 (5)	0.2083 (4)	0.10168 (12)	0.0290 (7)	
H6B	−0.001 (11)	0.317 (3)	0.101 (3)	0.05 (2)*	
H6A	−0.030 (11)	0.173 (10)	0.1268 (10)	0.042 (18)*	

### (Na3_Li_2SO4_6H2O-293K) Atomic displacement parameters (Å^2^)

**Table:**

	*U*^11^	*U*^22^	*U*^33^	*U*^12^	*U*^13^	*U*^23^
Li1	0.027 (4)	0.027 (4)	0.025 (7)	0.014 (2)	0.000	0.000
Na1	0.0298 (9)	0.0331 (10)	0.0285 (9)	0.0167 (8)	0.0058 (7)	0.0065 (7)
S1	0.0188 (6)	0.0188 (6)	0.0127 (8)	0.0094 (3)	0.000	0.000
S2	0.0173 (6)	0.0173 (6)	0.0161 (9)	0.0087 (3)	0.000	0.000
O1	0.0312 (17)	0.0265 (16)	0.0266 (16)	0.0185 (14)	−0.0024 (12)	−0.0045 (12)
O2	0.0261 (17)	0.0360 (17)	0.0297 (15)	0.0189 (14)	0.0011 (12)	0.0040 (13)
O3	0.048 (3)	0.048 (3)	0.013 (3)	0.0242 (15)	0.000	0.000
O4	0.029 (2)	0.029 (2)	0.017 (3)	0.0146 (11)	0.000	0.000
O5	0.0265 (16)	0.0226 (15)	0.0271 (14)	0.0118 (13)	0.0003 (11)	−0.0005 (10)
O6	0.0351 (19)	0.0276 (18)	0.0243 (15)	0.0156 (16)	0.0013 (12)	0.0008 (13)

### (Na3_Li_2SO4_6H2O-293K) Geometric parameters (Å, º)

**Table:**

Li1—O3	1.920 (15)	Na1—O4^v^	2.671 (5)
Li1—O5^i^	1.953 (6)	Na1—Na1^iv^	3.702 (5)
Li1—O5^ii^	1.953 (6)	Na1—Na1^vi^	3.702 (5)
Li1—O5^iii^	1.953 (6)	S1—O3	1.443 (6)
Li1—Na1^ii^	3.775 (8)	S1—O2	1.477 (3)
Li1—Na1^iii^	3.775 (8)	S1—O2^vii^	1.477 (3)
Li1—Na1^i^	3.775 (8)	S1—O2^viii^	1.477 (3)
Na1—O2	2.354 (4)	S2—O4	1.479 (6)
Na1—O6^iv^	2.372 (4)	S2—O1^ix^	1.480 (3)
Na1—O6	2.392 (4)	S2—O1^x^	1.480 (3)
Na1—O5	2.431 (4)	S2—O1	1.480 (3)
Na1—O1	2.481 (4)		
			
O3—Li1—O5^i^	110.3 (4)	O1—Na1—Na1^iv^	142.12 (9)
O3—Li1—O5^ii^	110.3 (4)	O4^v^—Na1—Na1^iv^	46.12 (9)
O5^i^—Li1—O5^ii^	108.6 (4)	O2—Na1—Na1^vi^	149.12 (10)
O3—Li1—O5^iii^	110.3 (4)	O6^iv^—Na1—Na1^vi^	85.24 (9)
O5^i^—Li1—O5^iii^	108.6 (4)	O6—Na1—Na1^vi^	38.80 (10)
O5^ii^—Li1—O5^iii^	108.6 (4)	O5—Na1—Na1^vi^	79.04 (11)
O3—Li1—Na1^ii^	126.13 (16)	O1—Na1—Na1^vi^	122.00 (10)
O5^i^—Li1—Na1^ii^	118.8 (5)	O4^v^—Na1—Na1^vi^	46.12 (9)
O5^ii^—Li1—Na1^ii^	34.45 (19)	Na1^iv^—Na1—Na1^vi^	60.0
O5^iii^—Li1—Na1^ii^	74.2 (2)	O2—Na1—Li1^xi^	72.81 (11)
O3—Li1—Na1^iii^	126.13 (16)	O6^iv^—Na1—Li1^xi^	167.34 (12)
O5^i^—Li1—Na1^iii^	74.2 (2)	O6—Na1—Li1^xi^	104.87 (13)
O5^ii^—Li1—Na1^iii^	118.8 (5)	O5—Na1—Li1^xi^	27.04 (10)
O5^iii^—Li1—Na1^iii^	34.45 (19)	O1—Na1—Li1^xi^	75.20 (18)
Na1^ii^—Li1—Na1^iii^	88.8 (2)	O4^v^—Na1—Li1^xi^	98.10 (19)
O3—Li1—Na1^i^	126.13 (16)	Na1^iv^—Na1—Li1^xi^	142.67 (16)
O5^i^—Li1—Na1^i^	34.45 (19)	Na1^vi^—Na1—Li1^xi^	105.96 (7)
O5^ii^—Li1—Na1^i^	74.2 (2)	O3—S1—O2	109.90 (16)
O5^iii^—Li1—Na1^i^	118.8 (5)	O3—S1—O2^vii^	109.90 (16)
Na1^ii^—Li1—Na1^i^	88.8 (2)	O2—S1—O2^vii^	109.04 (16)
Na1^iii^—Li1—Na1^i^	88.8 (2)	O3—S1—O2^viii^	109.90 (16)
O2—Na1—O6^iv^	94.56 (13)	O2—S1—O2^viii^	109.04 (16)
O2—Na1—O6	171.91 (15)	O2^vii^—S1—O2^viii^	109.04 (16)
O6^iv^—Na1—O6	87.53 (17)	O4—S2—O1^ix^	109.82 (15)
O2—Na1—O5	94.58 (13)	O4—S2—O1^x^	109.82 (15)
O6^iv^—Na1—O5	162.10 (14)	O1^ix^—S2—O1^x^	109.13 (15)
O6—Na1—O5	85.57 (14)	O4—S2—O1	109.82 (15)
O2—Na1—O1	88.07 (14)	O1^ix^—S2—O1	109.12 (15)
O6^iv^—Na1—O1	104.17 (14)	O1^x^—S2—O1	109.12 (15)
O6—Na1—O1	83.84 (12)	S2—O1—Na1	131.4 (2)
O5—Na1—O1	91.50 (13)	S1—O2—Na1	126.6 (2)
O2—Na1—O4^v^	103.02 (15)	S1—O3—Li1	180.0
O6^iv^—Na1—O4^v^	85.33 (14)	S2—O4—Na1^xii^	126.84 (13)
O6—Na1—O4^v^	84.92 (14)	S2—O4—Na1^xiii^	126.84 (13)
O5—Na1—O4^v^	77.62 (14)	Na1^xii^—O4—Na1^xiii^	87.75 (19)
O1—Na1—O4^v^	164.92 (11)	S2—O4—Na1^i^	126.84 (13)
O2—Na1—Na1^iv^	101.59 (12)	Na1^xii^—O4—Na1^i^	87.75 (19)
O6^iv^—Na1—Na1^iv^	39.20 (10)	Na1^xiii^—O4—Na1^i^	87.75 (19)
O6—Na1—Na1^iv^	84.96 (9)	Li1^xi^—O5—Na1	118.5 (3)
O5—Na1—Na1^iv^	123.52 (10)	Na1^vi^—O6—Na1	102.00 (15)

Symmetry codes: (i) *x*+1/3, *x*−*y*+2/3, *z*+1/6; (ii) −*y*+4/3, −*x*+2/3, *z*+1/6; (iii) −*x*+*y*+1/3, *y*−1/3, *z*+1/6; (iv) −*x*+*y*, −*x*, *z*; (v) −*y*+2/3, −*x*+1/3, *z*−1/6; (vi) −*y*, *x*−*y*, *z*; (vii) −*y*+1, *x*−*y*, *z*; (viii) −*x*+*y*+1, −*x*+1, *z*; (ix) −*x*+*y*, −*x*+1, *z*; (x) −*y*+1, *x*−*y*+1, *z*; (xi) −*y*+2/3, −*x*+4/3, *z*−1/6; (xii) −*x*+*y*+1/3, *y*+2/3, *z*+1/6; (xiii) −*y*+1/3, −*x*+2/3, *z*+1/6.

## References

[bb1] Brandenburg, K. (2017). *DIAMOND*. Crystal Impact GbR, Bonn, Germany.

[bb2] Bruker (2009). *TOPAS*. Bruker AXS, Karlsruhe, Germany.

[bb3] Coppens, P. (1970). *Crystallographic Computing*, edited by F. R. Ahmed, S. R. Hall & C. P. Huber, pp. 255–270. Copenhagen: Munksgaard.

[bb4] Filippov, V. K. & Kalinkin, A. M. (1989). *Zh. Neorg. Khim.* **32**, 215–217.

[bb5] Fugel, M., Malaspina, L. A., Pal, R., Thomas, S. P., Shi, M. W., Spackman, M. A., Sugimoto, K. & Grabowsky, S. (2019). *Chem. Eur. J.* **25**, 6523–6532. 10.1002/chem.201806247 30759315

[bb6] Groth, P. (1908). *Chemische Krystallographie – Die Anorganischen Oxo- und Sulfosalze*, Vol. 2. Leipzig: Verlag Wilhelm Engelmann.

[bb7] Ji, Z.-Y., Peng, J.-L., Yuan, J.-S., Li, D.-C. & Zhao, Y.-Y. (2015). *Fluid Phase Equilib.* **397**, 81–86.

[bb8] Kaminskii, A. A., Bohatý, L., Becker, P., Held, P., Eichler, H. J. & Rhee, H. (2007). *Phys. Stat. Sol.* **1**, R16–R17.

[bb9] Kaminskii, A. A., Bohatý, L., Becker, P., Held, P., Rhee, H., Eichler, H. J. & Hanuza, J. (2009). *Laser Phys. Lett.* **6**, 335–350.

[bb10] Klevtsova, R. F., Glinskaya, L. A. & Klevtsov, P. V. (1988). *Kristallografiya*, **33**, 1380–1386.

[bb11] Mitscherlich, E. (1843). *Ann. Phys. Chem.* **134**, 468–472.

[bb12] Parsons, S., Flack, H. D. & Wagner, T. (2013). *Acta Cryst.* B**69**, 249–259. 10.1107/S2052519213010014 PMC3661305 23719469

[bb13] Scacchi, A. (1867). *Atti della Accademia delle Scienze Fisiche e Matematiche di Napoli*, **3**, 25–31, 636–641.

[bb14] Sheldrick, G. M. (2008). *Acta Cryst.* A**64**, 112–122. 10.1107/S0108767307043930 18156677

[bb15] Sheldrick, G. M. (2015). *Acta Cryst.* C**71**, 3–8.

[bb16] Sohr, J., Voigt, W. & Zeng, D. (2017). *J. Phys. Chem. Ref. Data*, **46**, 1–221.

[bb17] Stoe & Cie (2015). *X-AREA* and *X-RED32*. Stoe & Cie, Darmstadt, Germany.

[bb18] Sugimoto, K., Dinnebier, R. E. & Schlecht, T. (2006). *J. Appl. Cryst.* **39**, 739–744.

[bb19] Traube, H. (1894). *N. Jahrb. Mineral.* pp. 185–195.

[bb20] Westrip, S. P. (2010). *J. Appl. Cryst.* **43**, 920–925.

